# Integrative expression network analysis of microRNA and gene isoforms in sacred lotus

**DOI:** 10.1186/s12864-020-06853-y

**Published:** 2020-06-25

**Authors:** Yue Zhang, Razgar Seyed Rahmani, Xingyu Yang, Jinming Chen, Tao Shi

**Affiliations:** 1 Key Laboratory of Aquatic Botany and Watershed Ecology, Wuhan Botanical Garden, Chinese Academy of Sciences, Wuhan, 430074 China; 2 Center of Conservation Biology, Core Botanical Gardens, Chinese Academy of Sciences, Wuhan, 430074 China; 3 University of Chinese Academy of Sciences, Beijing, 100049 China; 4 Department of Plant Biotechnology and Bioinformatics, Ghent University, Ghent, Belgium; 5 Wuhan Institute of Landscape Architecture, Wuhan, China

**Keywords:** microRNA, Transcript isoforms, Co-expression network, Sacred lotus

## Abstract

**Background:**

Gene expression is complex and regulated by multiple molecular mechanisms, such as miRNA-mediated gene inhibition and alternative-splicing of pre-mRNAs. However, the coordination of interaction between miRNAs with different splicing isoforms, and the change of splicing isoform in response to different cellular environments are largely unexplored in plants. In this study, we analyzed the miRNA and mRNA transcriptome from lotus (*Nelumbo nucifera*), an economically important flowering plant.

**Results:**

Through RNA-seq analyses on miRNAs and their target genes (isoforms) among six lotus tissues, expression of most miRNAs seem to be negatively correlated with their targets and tend to be tissue-specific. Further, our results showed that preferential interactions between miRNAs and hub gene isoforms in one coexpression module which is highly correlated with leaf. Intriguingly, for many genes, their corresponding isoforms were assigned to different co-expressed modules, and they exhibited more divergent mRNA structures including presence and absence of miRNA binding sites, suggesting functional divergence for many isoforms is escalated by both structural and expression divergence. Further detailed functional enrichment analysis of miRNA targets revealed that miRNAs are involved in the regulation of lotus growth and development by regulating plant hormone-related pathway genes.

**Conclusions:**

Taken together, our comprehensive analyses of miRNA and mRNA transcriptome elucidate the coordination of interaction between miRNAs and different splicing isoforms, and highlight the functional divergence of many transcript isoforms from the same locus in lotus.

## Background

The genetic central dogma only illustrates a portion of gene regulation since gene expression regulation is a multi-layer mechanism involving more processes such as alternative splicing of pre-mRNAs, and non-coding RNA regulation. Among non-coding RNAs, microRNAs (miRNAs) are one of the most important groups that can interact with the gene at the RNA level. In plants, microRNAs (miRNAs) are a class of small endogenous single-stranded noncoding RNAs ranging from 18 to 24 nucleotides in length [1]. The primary miRNAs are derived from MIRNA genes transcribed by RNA polymerase II and further processed by dicer-like 1 (DCL1) to yield the precursor-miRNAs (pre-miRNAs) [2, 3]. The pre-miRNAs are later diced into short miRNA duplexes containing one or two mature miRNAs. Given that many miRNAs are tissue or species-specific, much research has been conducted to explore the function of plant miRNAs indicating that the plant miRNAs play key roles in response to plant development, abiotic and biotic stresses through regulating their target genes [4–6].

The silencing or translational repression of genes containing miRNA binding sites is a post-transcriptional mechanism of gene regulation [7]. Several studies have suggested that a substantial amount of the miRNA targets are transcription factors or stress-response factors that are essential for biological processes. Lacking miRNA regulation, plants would face multiple developmental defects in many critical developmental stages [8–10]. High throughput small RNA sequencing is efficient and accurate to elucidate miRNA expression profiles and has been employed in many plant studies to uncover the roles of miRNAs in organ growth and response to the environmental stimuli [11–14]. Through differential expression analyses, studies found many differentially expressed miRNAs that participate in different processes and pathways such as auxin signal transduction during pollination of maize silks [15] and root development in Arabidopsis [16, 17].

RNA alternative splicing (AS) is another important post-transcriptional regulation mechanism, producing diverse transcript isoforms encoded by the same genes [18]. With the widespread application of full-length transcriptome sequencing technology, plenty of isoforms produced by alternative splicing events were identified in plants [19–21]. The structure variation in transcript isoforms can often result in proteins with altered physical characteristics and molecular functions [22]. In some cases, the presence or absence of the miRNA binding site in the isoform determines the possibility of its silencing by a complementary miRNA, allowing some isoforms to escape from being targeted due to lack of the miRNA binding site. This phenomenon of miRNA escaping through mRNA splicing has been identified in cotton and maize, indicating the gene regulation which can be interplayed by both miRNAs and AS [23, 24]. Nowadays, investigations on the regulated network of miRNA-mRNA interactions have been carried out in some model plants, such as Arabidopsis and rice, to identify the key genes related to abiotic stress [25, 26]. These studies focused on the regulation of miRNA on target gene expression, but the influence of miRNAs on the co-expression network of different splicing isoforms calls for further investigation in the plant. Besides, our understanding of expression and functional divergence of isoforms in response to different developmental and growth factors is impeded by the paucity of relevant case studies in plants [19–22].

Lotus or sacred lotus (*Nelumbo nucifera*) is an important aquatic plant with utility in horticulture, landscape, and medicine, which is widely cultivated in Asia. Our previous deep-sequencing of miRNAs in six different tissue samples uncovers the evolution and diversity of miRNAs in lotus [27]. Meanwhile, by combining the full-length transcriptome sequencing and RNA-seq dataset of lotus, we also identified a large amount of AS events showing tissue-specific regulatory manner [28]. However, the interactions between miRNAs and targets at the isoform level, and the impact of miRNAs on target gene and isoform expression profiles are still unclear. In this study, comparative analyses of expression profiles between miRNAs and their target genes (and isoforms) were carried out, aiming to explore the spatial and temporal regulation of miRNAs in lotus. Combining the identified full-length isoforms and small RNA-seq data, we also comprehensively investigated the interactions between miRNAs and their target isoforms by WGCNA (weighted gene co-expression network analysis) to uncover the impact of miRNAs on the expression and function of their target isoforms.

## Results

### Identification of microRNAs in the new lotus reference genome

**Fig. 1:**
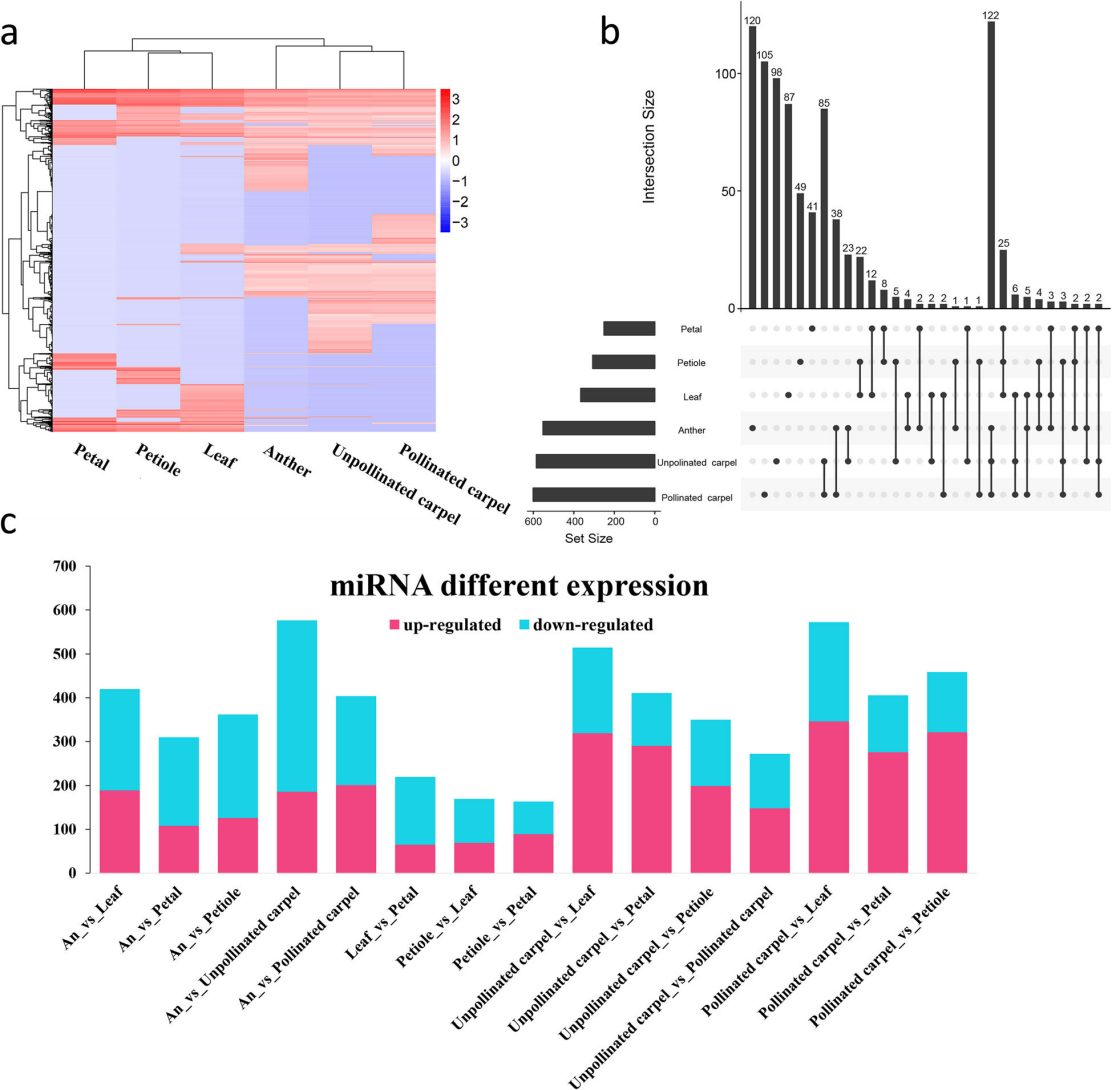
Summary of the miRNA expression. **a** A global view of the expression profile of all mature miRNAs in six tissues. **b** The UpSet plot summarizes the presence of mature miRNA in six tissues. The bottom left horizontal bar graph shows the total number of mature miRNA in per tissue. The circles in each panel’s matrix represent the unique and common parts in Venn diagram sections (unique and overlapping mature miRNAs). Connected circles indicate a certain intersection of mature miRNAs between tissues. The top bar graph in each panel summarizes the number of mature miRNAs for each unique or overlapping combination. **c** The bar plot of differentially expressed miRNAs between six samples. The red is the up-regulated miRNA and the blue is the down-regulated miRNA

To obtain a more comprehensive miRNA profile, we reanalyzed sRNA-seq datasets from six lotus tissues including leaf, petiole, petal, anther, unpollinated carpel and pollinated carpel, based on an updated miRbase database and an improved chromosome-level genome assembly of lotus. A total of 22.2 million filtered reads were mapped to the known miRNAs in miRBase (Table 1). The ratio of filtered high-quality reads mapped to the miRBase is 0.33%, i.e. a total of 50,866 reads aligned to the reference genome (nelumbo.biocloud.net) (Table 1) [29]. After merging with previous lotus miRNAs [27] and removing the redundant (overlapping) hairpin loci, a total of 1103 potential mature miRNA and 104 miRNA-star (the opposite strand of miRNA from dsRNA) sequences were identified, and these miRNAs are produced by 1416 pre-miRNAs (hairpin loci) (Fig. 1a)(Additional file 2: Table S1 and S2). The number of detected mature miRNAs is less than pre-miRNAs because many pre-miRNAs from distinct duplicate MIRNA genes can produce identical (short) mature miRNA sequence, which was also reported in other species (http://mirbase.org). Comparing the origin of the pre-miRNAs with transposable elements (TE) region in genome, 623 (43.99%) pre-miRNAs were found to be TE-related, suggesting that a substantial number of the miRNAs originate from TEs [30, 31]. In addition, only 444 (40.25%) of those mature miRNAs were identified as miRNA in the previous analysis [27]. Furthermore, 235 (19.46%) of miRNAs were known sequences in miRBase database and 528 (43.74%) are novel miRNAs identified in this study. Among these currently identified novel miRNAs, 348 (65.9% of novel) are potentially produced by TE-related MIRNA-likes genes. By length, the 24 bp miRNAs are the most abundant while 388 (58.43%) of which are TE-related, supporting that the emerging of novel miRNAs from TEs [32, 33] (Additional file 1: Fig. S1). Furthermore, we observed that the frequency of each nucleobase (A, U, C and G) in the miRNAs was close to 25% (Additional file 1: Fig. S2). However, we also determined the frequency of the base of the mature miRNAs, the result showed that the 20 bp, 21 bp, and 22 bp miRNAs preferentially start with ‘U’ at the first base (46.96, 55.37, and 61.22%, respectively) (Additional file 1: Fig. S3), while 24 bp miRNAs preferred ‘A’ (58.5%). Comparing with miRNA’s first nucleotide bias analysis in other species, we found the bias tendency in 21 bp, 22 bp and 24 bp miRNAs is similar to *Camellia japonica* [34], pomegranate [35].

**Table 1:** Summary of high-quality reads mapped to miRBase

Sample	High-quality reads	Reads with at least one alignment in miRBase	Reads without alignment in miRBase
Anther	6,826,041	11,337 (0.17%)	6,814,704 (99.83%)
Unpollinated carpel	7,164,301	10,947 (0.15%)	7,153,354 (99.85%)
Pollinated carpel	8,943,060	13,735 (0.15%)	8,929,325 (99.85%)
Petiole	1,472,529	6118 (0.42%)	1,466,411 (99.58%)
Leaf	1,707,006	7964 (0.47%)	1,699,042 (99.53%)
Petal	1,518,064	5984 (0.39%)	1,512,080 (99.61%)

### Expression dynamics of miRNAs and their target genes across different tissues

Through differential regulation in different tissues or developmental stages, miRNAs play pivotal roles in diverse biological processes including development [4, 5]. To gain insight into the miRNA expression pattern across different lotus tissues, we first performed hierarchical clustering on the expression data from our identified mature miRNAs (Fig. 1a). Interestingly, we found that the majority of miRNAs are preferentially expressed in specific tissues. Only 110 miRNAs are commonly expressed in all tissues; carpel has the most specific miRNAs, followed by anther (Fig. 1b). A total of 1003 differentially expressed miRNAs were identified. We identified differentially expressed miRNA in other tissues relative to pollinated carpel, and the up-regulated miRNAs outnumber the down-regulated miRNA in the pollinated carpel, indicating that there could be intensive activation of miRNAs in carpel after pollination (Fig. 1c).

**Fig. 2:**
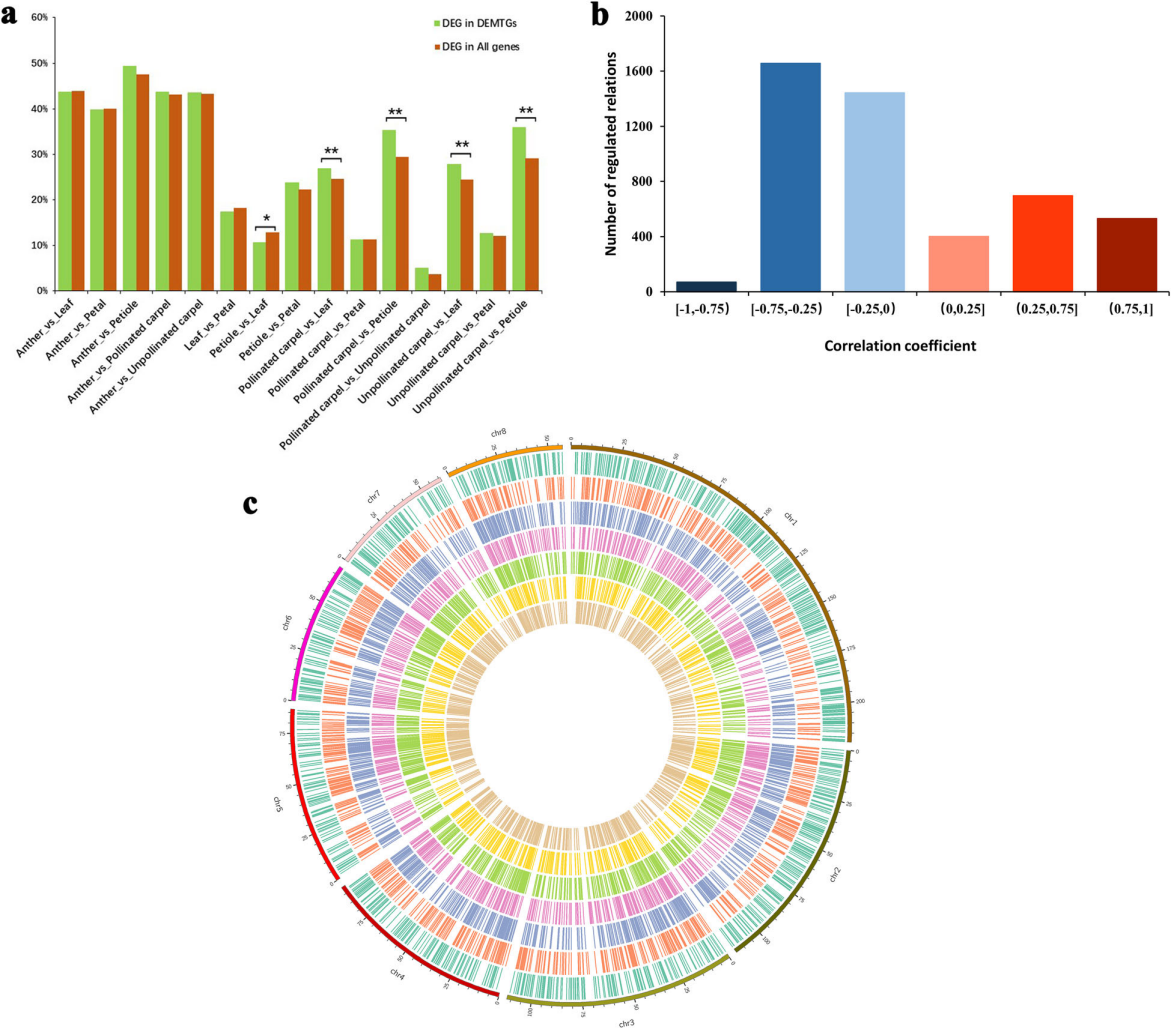
Relationship between miRNAs and their target genes. **a**. Impact of differentially expressed miRNAs (DEM) on the expression of their target genes. Green (DEMTGs): the proportion of differentially expressed genes (DEGs) as targets of differentially expressed miRNAs; brown (DEGs): the proportion of DEGs in the genome background. **b** The distribution of the number of miRNA-target pairs according to Pearson’s correlation coefficient of target gene expression and miRNA expression. **c** The CIRCOS plot of the distribution of pre-miRNAs and miRNA target genes in chromosome 1–8. Seven circles from the outside to the inside show the chromosomal distribution of pre-miRNAs, miRNA target genes in anther, miRNA target genes in leaf, miRNA target genes in petal, miRNA target genes in petiole, miRNA target in unpollinated carpel and miRNA target gene in pollinated carpel, respectively

The Pearson correlation coefiicients among gene expression profiles generated by the RNA-seq analysis of biological replicates suggested the high reproducibility between replicates (ave *r* > 0.859, all *p*-value < 0.0001) (Additional file 1: Fig. S4). To explore the expression pattern of miRNA target genes among different tissues, pairwise comparisons of these six samples were conducted to identify differentially expressed genes (DEGs). A total of 28,701 DEGs were identified by using the edgeR package. The comparison between anther and petiole shows the most DEGs, whereas the comparison between pollinated carpel and unpollinated carpel reveals the least DEGs (Fig. 2a). To explore whether differentially expressed miRNAs might escalate the expression difference of their target genes between tissue samples, we calculated the proportion of DEGs in the target genes of those differentially expressed miRNAs (DEMTGs) and compared it to DEGs in the genome background. The comparison between anther and petiole also exhibits the highest percentage 49.26% (740) of DEMTGs, while the comparison in pollinated carpel and unpollinated carpel has the lowest percentage of 5.07% (18) (Fig. 2a). The proportion of DEGs in DEMTGs is generally higher than that of DEGs in all genes for most between-tissue comparisons, especially in the comparison between carpel and leaf, between carpel and petiole (χ^2^ test, all *p*-value< 0.01), except for the comparison between petiole and leaf. This indicates that the differentially expressed miRNAs among tissues might influence the expression of their targeted gene to some extent.

**Fig. 3:**
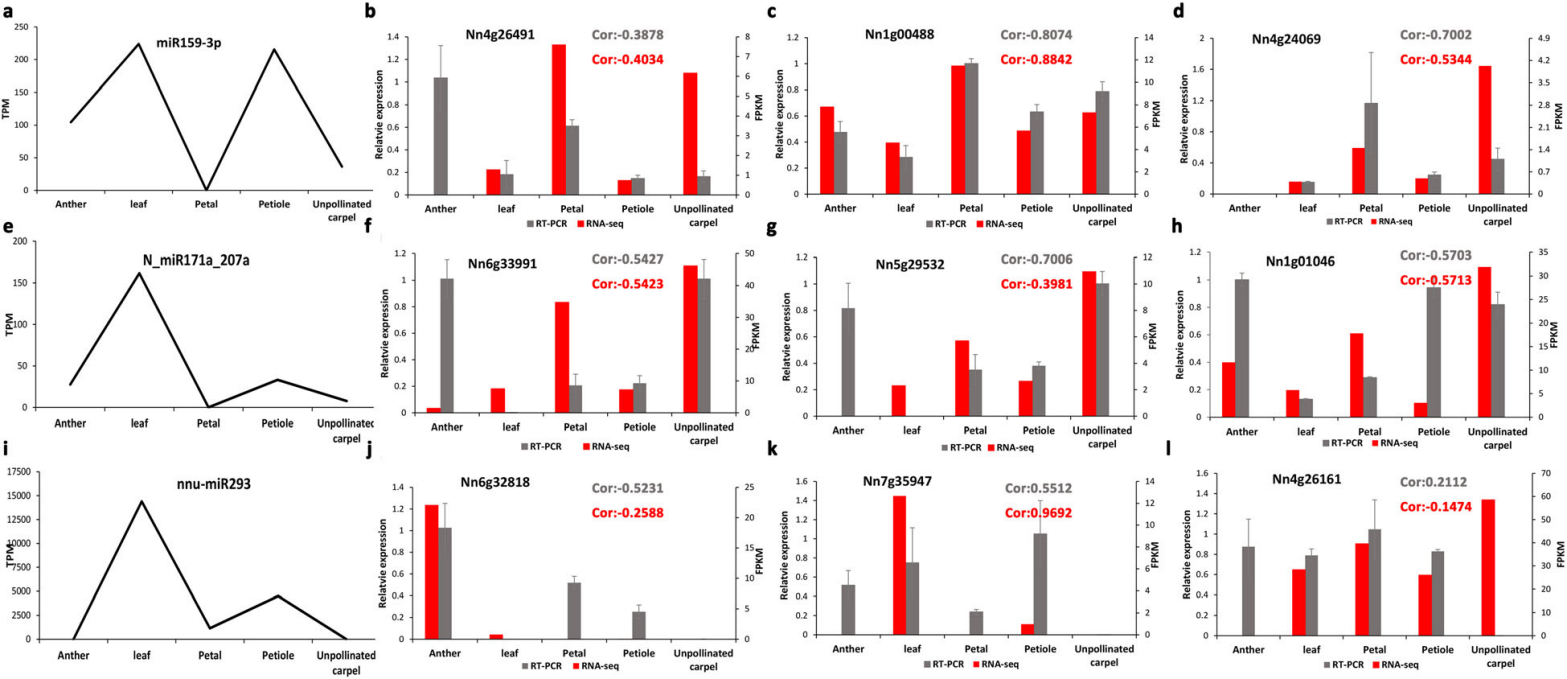
Expression profile of several selected miRNAs and targeted genes. The expression of miRNAs is shown in the line graph on the left column. The expression of the targeted gene is shown in the bar plot in the right three columns. The correlation coefficient between miRNA and the targeted gene is shown. Gray: RT-PCR result; red: RNA-seq result. The a-d figures show “miR159-3p” and its corresponding targeted gene. The e-h figures show “N_miR171a_207a” and its corresponding targeted gene. The i-l figures show “nnu-miR293” and its corresponding targeted gene

To further explore how intensively the expression pattern of target genes was influenced by the miRNA, the expression correlation analyses between target genes and miRNAs across different tissue samples were carried out (Additional file 2: Table S3). In this study, the correlation coefficient (*r*) between miRNA and target gene is divided into six levels: strong negative correlation (− 1 to − 0.75), intermediate negative correlation (− 0.75 to − 0.25), weak negative correlation (− 0.25 to 0), weak positive correlation (0 to 0.25), intermediate positive correlation (0.25 to 0.75) and strong positive correlation (0.75 to 1). The result showed a substantial bias toward negative correlations such that the negative correlations are about double comparing with positive correlations (Fig. 2b). The intermediate negative correlations and weak negative correlations are prevalent, and the strong negative correlations are the least, suggesting that miRNAs still mainly repress their target genes (Fig. 2b). We further investigated the expression level of targeted genes in different samples, which revealed that the expression of targeted genes is varied between samples possibly due to the expression difference of miRNAs between samples (Fig. 2c). To validate the potential regulation of miRNA targets, we randomly selected 15 miRNA targeted genes to perform real-time qPCR experiments. We carried out correlation analyses between miRNAs expression and RT-PCR result of target genes and compared with corresponding correlation obtained from RNA-seq expression. Among 15 pairs of correlation between miRNA and target genes, 12 pairs (80%) showed the negative correlation based on both results from RT-PCR and RNA-seq, further revealing the complex regulatory relationships between miRNAs and target genes. (Fig. 3, Additional file 1: Fig. S5).

### Differentially expressed miRNA and their target isoforms

Taking advantage of transcript isoform analyses from our previous study [28], we further analyzed the miRNA-target isoforms instead of genes. A total of 10,345 unique target isoforms were predicted (Additional file 2: Table S4). Most target isoforms (8850, 85.54%) contain only one miRNA target site; a small portion of isoforms (847, 8.18%) contain two miRNA target sites; the rest contain more than two miRNAs target sites (Additional file 1: Fig. S6a). Notably, the isoforms ‘Nn8g40904.1’ and ‘Nn8g40902.1’ can be bound by many miRNAs, with 38 and 31 homologous miRNAs from the family miR169, respectively. We also calculated the number of regulatory miRNAs per target gene, and expectedly the distributions of the number of regulatory miRNAs for miRNA-targeted genes and miRNA-targeted isoforms are similar (Additional file 1: Fig. S6b). Not all miRNA-targeted genes have all their corresponding isoforms being targeted by miRNAs--there are only 1637 target genes having all of their isoforms targeted by the specific miRNAs, such as ‘Nn3g21300’ (*AFB3*) (Additional file 1: Fig. S7), whereas there are 2449 target genes with only a portion of their isoforms being targeted, such as ‘Nn3g21564’ (Additional file 1: Fig. S7). We further compared the expression level of miRNA-targeted isoforms and non-miRNA-targeted isoforms from the same genes. Interestingly, we found that miRNA targeted isoforms tend to have significantly higher expression level in all investigated tissue samples, suggesting that the isoforms containing miRNA binding sites are under miRNA-mediated expression tuning and buffering likely because of their high expression level representing the functional importance (Additional file 1: Fig. S8). The most miRNA target sites in gene bodies are on coding regions (CDSs) (74.76%), whereas the 5′-UTRs (9.59%) and 3′-UTRs (15.65%) regions have fewer target sites by miRNAs. Given that a substantial number of TE-related miRNAs were found in this study, it is essential to know if they also have a regulatory role in gene expression. We found that 43.57% of TE-related miRNAs have a target gene while 50.28% of non-TE-related miRNAs have a target gene, suggesting that the TE-related miRNAs also play an important role in regulating genes (Additional file 2: Table S2, S4).

To understand the biological functions of miRNAs, especially those tissue-specific miRNAs, functional annotation based on gene ontology (GO) was used. We found that only 1979 out of 4086 miRNA target genes were annotated by GO categories (Additional file 2: Table S5; Additional file 1: Fig. S9). Among the most significantly enriched GO terms of target genes are “endonuclease activity,” “regulation of transcription, DNA-templated” and “Cul4-RING ubiquitin ligase complex,” indicating that the genes targeted by miRNA can regulate numerous key processes and many belonging to transcription factors [36, 37]. The specific miRNA may regulate specific genes being crucial in the different developmental stages, and therefore GO functional enrichment analysis was conducted for six samples (Additional file 1: Fig. S10). In anther, the most enriched GO terms are related to plant reproductive processes such as “microtubule organizing center,” “auxin-activated signaling pathway” and “endonuclease activity.” In petiole, the miRNA target genes are enriched in “chloroplast stromal thylakoid” and “leaf development.” Both in the pollinated and unpollinated carpel, the most enriched GO terms are the same, i.e. “sepal development,” “regulation of anthocyanin biosynthetic process” and “miRNA binding.” These results collectively revealed that the functions of the miRNA target genes are closely related to the tissue-specification.

### Functional differentiation of isoforms in the co-expression networks

**Fig. 4:**
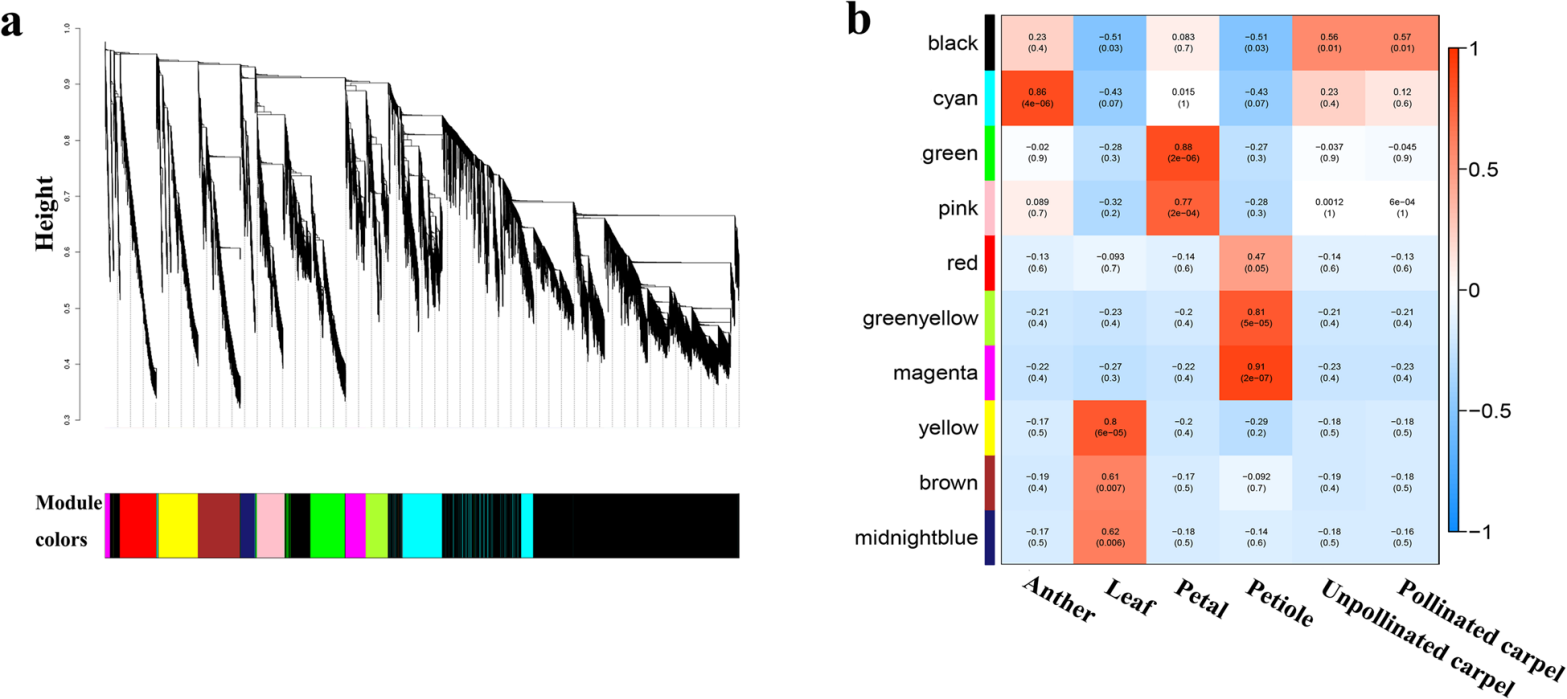
The co-expression network of filtered isoforms. **a**. Hierarchical cluster tree and color bands indicating 9 modules identified by weighted isoforms co-expression network analysis. **b**. The analysis of module-trait correlation. Each row represents a module and each column represents a specific sample. Each cell at the row-column intersection is color-coded by correlation according to the color legend. Each cell has two values: the up value is the correlation coefficient between the module genes and sample; the down value is the *p*-value

It is often assumed that the tightly connected genes in the co-expression network are likely participating in the same biological process, and therefore it provides a means to identify functional divergence between isoforms. Here, we performed WCGNA at the transcript isoform level. We found that some isoforms are exhibiting dramatic expression differences among different tissues. To explore the potential function of miRNA-targeted isoforms in different tissue, we first performed a hierarchical clustering analysis of total isoforms, and we found that a substantial portion of isoforms showed strong tissue-specificity (Additional file 1: Fig. S11). After filtering out the lowly expressed (FPKM < 0.1) and universally expressed (C.V. of FPKMs across six tissue samples < 2) isoforms, 56,583 isoforms were retained to construct a co-expression network by using WGCNA. A total of 10 modules were defined as clusters of major tree branches (Fig. 4a), with the module size ranging from 766 to 13,309, and isoforms within the same cluster have high correlation coefficients among each other (Additional file 2: Table S6, Fig. 4b). We further investigated correlations between the tissues and the 10 co-expression modules. Most modules are significantly (*p* < 0.05) correlated with single tissue, except that the black module is significantly correlated with both pollinated carpel and unpollinated carpel. Basically, isoforms in each module are over-represented in the corresponding tissue, and the 150 candidate hub isoforms for each module were assigned (Additional file 1: Fig. S12). The correlation analysis between the modules revealed that black, cyan, green and pink module, which are significantly correlated with the three floral organs, also have high correlation among each other, proving the accuracy of the module clustering and the homology of differentiated floral organs (Additional file 1: Fig. S13). Because the leaf and petiole are both vegetative tissues, six modules are significantly correlated with leaf or petiole, respectively. To explore the influence of miRNAs on the co-expression network of isoforms, we calculated the content of miRNA-targeted isoforms and the number of hub isoforms in every module (Additional file 1: Fig.S14). Moreover, our further χ^2^ test analysis at module level revealed that only the proportion of isoforms in the brown modules being targeted by miRNAs (184/2260, 8.14%) is significantly lower than the corresponding proportion of isoforms in hubs (51/150, 34%) (χ^2^ test, *p* < 0.01) (Additional file 1: Fig. S14). This suggested that miRNAs preferentially target hub isoforms in the brown module, which is highly correlated with leaves.

The isoforms from the same gene are often translated into protein variants with different structures and, hence, performing different functions [22]. To understand the scale of functional differentiation among isoforms from the same gene, we identified isoforms that were assigned to different modules in the co-expression network. Interestingly, among 11,302 genes with multiple isoforms being assigned to modules, 3029 genes have their isoforms being assigned into different modules (GIDDM). Moreover, 464 of these GIDDMs were targeted by miRNAs. This supports that substantial genes with multiple isoforms show functional divergence between isoforms. For example, “Nn5g29774”, annotated as ‘responding to salt stress’, produce a total of 41 isoforms, and 18 of them were clustered into five modules, including 12 in cyan, three in red, one in pink, one in black and one in brown (Additional file 1: Fig. S15). Among these 18 isoforms belonging to different modules, and five of them were regulated by two miRNAs, one by nnu-miR200 and one by miR-1655-3p.

If the isoforms of the same gene are functionally divergent, we assume that these different isoforms might likely convert into different genes (duplicates) to play their independent functions during the long-term evolution. To validate this assumption, we searched the closest homologous isoform in rice and Arabidopsis, respectively, for each lotus isoform. After filtering out genes with only one isoform, the gene can be divided into three categories: different isoforms from the same lotus gene with their closest homologs being different genes in rice or Arabidopsis (I); different isoforms from the same lotus gene with their closest homologs being the same isoform from the same gene in rice or Arabidopsis (II); different isoforms from the same lotus gene with their closest homologs being different isoforms from the same gene in rice or Arabidopsis (III). The results show that the number of genes in category II is the most (Additional file 2: Table S7, Additional file 1: Fig. S16). However, interestingly, when only considered the GIDDM, the proportions of isoforms in category I, were largely increased by 10.3 and 9.8%, respectively in rice and Arabidopsis. The result further substantiates that different isoforms, belonging to different co-expression modules, from the same gene tend to evolve into more divergent sequence structures. Meanwhile, this shows that these isoforms were more likely to convert into different duplicate copies during long-term evolution.

### MiRNA-targeted isoforms in plant hormone signaling pathways

To further elucidate the functions of miRNAs and their target isoforms, we focused on the phytohormone pathways enrichment since they are essential in almost all biological processes in the plant. First, KEGG annotation found that a total of 397 miRNA target genes were assigned to 106 pathways. ‘Plant hormone signaling transduction’ was the third most enriched pathways and represented by 20 genes. These 20 genes are in auxin-, cytokinin-, gibberellin-, abscisic acid-, ethylene, brassinosteroid- and jasmonic acid-associated signaling pathways, targeted by 24 miRNAs (Additional file 1: Fig. S17). Among the 20 signaling genes, 16 of them were assigned to different modules in the co-expression network at isoform level and four were not assigned to any module, suggesting that miRNA target genes in the hormone pathways are mostly functionally relevant in different lotus tissues (Additional file 1: Fig. S18).

**Fig. 5:**
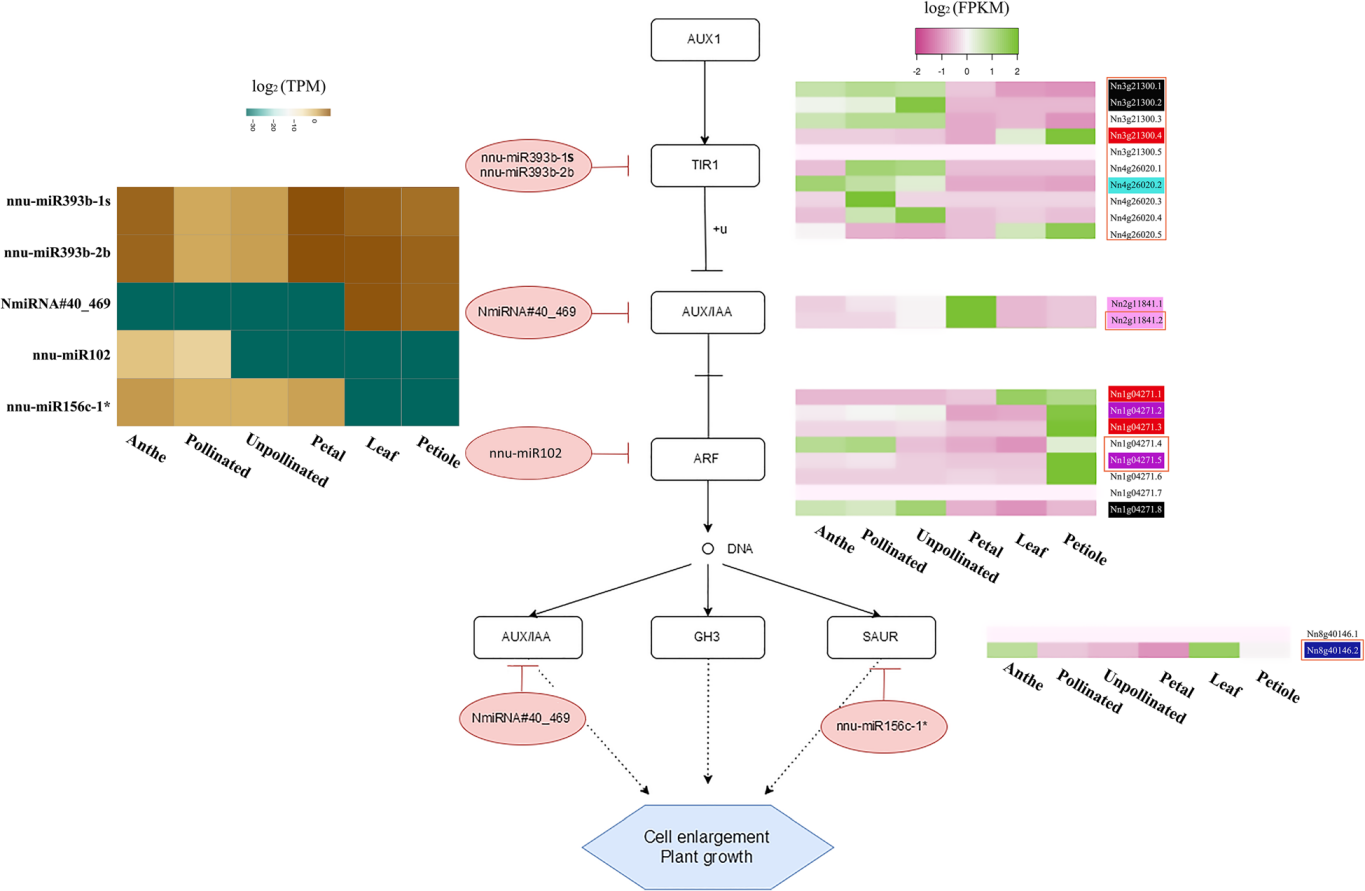
Correlation analysis of expression between miRNAs and auxin pathway genes in lotus

In auxin signaling pathways, the auxin receptor *TRANSPORT INHIBITOR RESPONSE1* (*TIR1*), the *auxin-responsive gene auxin/indole-3-acetic acid* (*AUX/IAA*), the *auxin response factor* (*ARF*) the *small auxin up RNA* (*SAUR*) are targeted by “nnu-miR393b-1 s”, “nnu-miR393b-2b”, “NmiRNA#40_469”, “nnu-miR102”,” nnu-miR156c-1*”. Combining the expression of miRNA and their target isoforms, the result revealed that the altered expression levels of targeted isoforms were not always negatively correlated with their corresponding miRNAs (Fig. 5). For example, the expression pattern of most isoforms from *TIR1* was almost negatively regulated by miRNAs (Fig. 5). However, the two isoforms of *TIR1*, “Nn3g21300.4″ and “Nn4g26020.5″, were highly expressed in leaf and petiole, same with the expressed pattern of the corresponding miRNAs “nnu-miR393b-1 s” and “nnu-miR393b-2b”. In another example, we found that the low expression of “nnu-miR102” in petiole might be associated with high expression of most targeted isoforms “Nn1g04271” (*ARF*) in petiole except for “Nn1g04271.8″ (Fig. 5). Meanwhile, there are four isoforms, transcribed by Nn1g04271, clustered into red and magenta modules highly correlated with petiole, suggesting the important regulatory relationship of nnu-miR102-*ARF* in auxin signaling of the petiole. In addition to the auxin signaling genes, similar regulatory relationship, and expression patterns in abscisic acid signaling were also observed (Additional file 1: Fig. S19). For example, the *phosphatase 2C* (*PP2C*) is targeted by five miRNAs, which are lowly expressed in the unpollinated carpel. Isoforms from one of two miRNA-targeted genes, “Nn6g35319”, are highly expressed in the unpollinated carpel, whose five isoforms were clustered into the black module. Nevertheless, the miRNA “nnu-miR8” and its targeted genes, homologous to serine/threonine-protein kinase *CTR1*, have high expression in anther, which, however, do not have a negative expression pattern between the miRNAs and target genes. Although we found many miRNAs and their target isoforms in plant hormone signaling pathways, their regulatory relationships are much more complex.

## Discussion

Gene expression is complicated and regulated by multiple mechanisms, such as non-coding RNAs and transcript splicing. Increasing pieces of evidence indicate that miRNAs play a vital role in plant growth and development by regulating their target genes [4]. Previously, we have identified a great number of miRNAs in six tissues of lotus, and also unveiled the evolutionary patterns for miRNA families with different ages [27]. Further, our other study on lotus transcript isoforms has been facilitated by full-length transcript sequencing using a combination of PacBio and Illumina [28]. The strategy of the combination of miRNAs and mRNA by deep sequencing has been successfully applied in many plant species, such as soybean [38], peanut [39] and cotton [40]. Therefore, in this study, we focused on how the expression of miRNAs in different lotus tissues influences the expression dynamics of their target genes and especially targeted isoforms. Currently, based on an updated miRbase database and our new version of lotus genome assembly, we identified 1207 unique mature miRNAs in these lotus tissues, which help us to discover some novel miRNAs which were missed by the previous study. Most of the novel identified miRNAs in our current study are related to transposable elements. Several studies found that many miRNAs, including *Ta*miR1123 that function in vernalization in wheat, 10 miRNAs in Arabidopsis and 38 in rice are derived from miniature inverted-repeat transposable elements (MITEs) [30, 41, 42]. As we found a great number of TE-related miRNA that can also have target genes in lotus, we demonstrated that TEs are an important source to give birth to novel functional miRNAs. Meanwhile, combining miRNA sequencing with the corresponding RNA-seq transcriptome profiles, our study revealed the importance of miRNA-mediated regulation in the growth of different lotus tissues. More interestingly, we elucidated the interactions between miRNA and different transcript isoforms. By building a weight isoform co-expression network, we evaluated the impact of miRNA on isoform expression pattern and uncovered the functional divergence of many isoforms originating from the same gene by partitioning into different co-expression modules. These findings will facilitate our current understanding of gene regulation by miRNAs and splicing isoforms in the plant.

In this study, most miRNAs are found to be preferentially accumulated in specific lotus tissues, suggesting that most miRNAs play more specialized roles during plant growth and development. In many case studies in plants, the miRNA sequences also appear to be tissue-specific, supporting that the specialized functions for most miRNAs [11, 43]. In our study, the tissue-specific miRNAs are preferentially enriched in the reproductive tissues, such as carpel and anther. Functional enrichment of miRNA-targeted genes showed that the tissue-specific miRNAs were important regulators in anther development, such as the formation of spindle fiber during mitosis. For example, the miR393 was identified to regulate *MPS1*, which regulates cell cycle function during anther development in cotton [44], and the homologous miRNA “nnu-miR393a” was also found to be specifically expressed in lotus anther, reflecting its conserved biological function. Besides, the miR172 family members, which regulate AP2, are involved in forming the sepal and petal primordia in *Arabidopsis* [45]. Several miR172 family members, such as “nnu-miR172b,” were specifically expressed in lotus anther to probably keep it from being developed into stamen petaloid. Our further miRNA differential expression analyses showed that there are more miRNAs induced in the pollinated carpel than in other tissues, indicating that miRNAs might be more active in tissues that undergo dramatic physiological changes, such as pollen-pistil interaction.

One novelty of our study is to investigate the relationship between miRNAs and their target isoforms because there are few studies focus on this aspect. Studies in human elucidated the interaction between miRNA and hub gene in a gene network [46, 47], yet not at isoforms levels. However, the intensive interactions between miRNAs and the brown module hub genes from the co-expression network in our current lotus study highlight the vital regulatory roles of miRNAs in the leaf. More interestingly, due to the different regions of isoforms from the same gene caused by alternative splicing or alternative transcriptional start site, we found some miRNAs can only target a portion of isoforms from the same gene while they miss the target for the other isoforms. Another novelty of our study is that we further explored the functional relevance of target genes between tissues and functional divergence of isoforms by building an isoform-based co-expression network. Meanwhile, the reason why we selected WGCNA for co-expression analysis is its diverse functions and maturity of data processing which has been successfully applied in many gene network and functional genomic studies [38, 48, 49]. Our findings revealed the case that some isoforms from the same gene appear to be divergent in the isoform co-expression network and selectively targeted by miRNAs due to their difference in sequence structure, suggesting the coordination of both structural and expression alteration during gene regulation. Our further homology-based analysis uncovered that those isoforms from the same gene, partitioned into different modules, also tend to have their homologs matching different genes in another plant species. We speculated that gene duplication and splicing isoforms might be interchangeable during the long-term evolution of plants. This phenomenon of ‘isoform-duplicate conversion’ is also found during vertebrate evolution including humans [50].

Given that several studies suggest that miRNA has emerged as key regulators of plant hormone response pathways by affecting their metabolism, distribution, and perception [16], we also focused on miRNA-targeted-isoform relationships in these pathways. It was shown that the miR393 regulates the lateral root development in *Arabidopsis* and leaf morphogenesis in cucumber by down-regulating the receptor *TIR1* during auxin signaling [51, 52], and the similar miRNA-target relationship was found in lotus, suggesting the conserved regulatory relationship during plant evolution. We also found that the *SAUR* gene related to auxin signaling pathway was targeted by miR156 (nnu-miR156c-1*), as this is also found in *Arabidopsis* [53]. Additionally, the *PPC2*, abscisic acid signal pathway genes, were found to be targeted by miR166 and involved in regulating the plant height in *Gossypium hirsutum* [54]. Our study found that the *PPC2* orthologous gene in lotus is targeted by five miRNAs, and the target isoforms were lowly expressed in the petiole, implying that the miR166-*PPC2* might participate in the regulation of plant height in lotus. As plant hormones have pivotal roles in plant development, these hormone-related miRNAs found in our study might provide an important genetic basis for future molecular genetic studies and manipulation.

## Conclusion

In the present study, we conducted systematic miRNA and mRNA transcriptome analyses in six lotus tissues and discovered more novel miRNAs. We showed that most miRNAs have tissue-specificity in expression and a negative correlation with the expression of their targeted genes. Meanwhile, the genes regulated by miRNA are involved in multiple biological processes, especially plant hormone response pathways. Our co-expression network at isoform level by using the WGCNA highlights the core regulatory role of miRNAs as the intensive interactions between miRNAs and hub isoforms. We also found that the isoforms from the same gene can be selectively targeted by miRNAs, and we further explored the functional divergence of these isoforms in both structure and expression. Collectively, this study on the interactions between the miRNAs and isoforms, and functional divergence of isoforms can facilitate our current understanding of the complexity of gene regulation in plants.

## Methods

### Plant material and RNA extraction

The tissues including leaf, petiole, petal and mature anther were harvested from *N. nucifera* var. ‘China Antique’ in Wuhan Botanical Garden, CAS (114^o^30’E, 30^o^60’N). The unpollinated and pollinated carpel were collected on a blooming day according to our previous study [27]. The samples were immediately frozen in liquid nitrogen and the total RNAs were extracted from each sample using RNAprep pure Plant Kit (TIANGEN). And the RNA was only used for RT-PCR validation experiments in this study. Meanwhile, the corresponding RNA-seq datasets from our previous study were downloaded from the NCBI Short Read Archive (SRA) accession number PRJNA503979 and PRJNA492157 [55].

### Small RNA-seq analysis

The small RNA-seq dataset was downloaded from the SRA accession number SRX1591010 [27]. The latest plant miRNA sequences were downloaded from miRbase 22.0 for miRNA annotation. For each sample, the high-quality reads without adaptors were filtered to retain sRNAs reads length from 18 bp to 29 bp. Before miRNA identification, the retained reads were further processed using the miRDeep2 [56]. After quality filtering, the Bowtie was used to map the high-quality reads to the reference genome (nelumbo.biocloud.net) with zero mismatches [29, 57]. The mapped small RNAs were guided by the known precursor miRNA dataset (miRbase 22.0) to identify potential miRNA precursors in the lotus genome, allowing the duplicate (hairpin) loci less than five. The hairpin structures and the aligned small RNAs were processed as described in the miRDeep-P package [58]. The overlapping (redundant) precursors were removed. Subsequently, we identified TE-related miRNAs as those pre-miRNAs overlapping with transposable elements in the lotus genome. To quantify the expression level of miRNA, the read counts were converted into TPM [59]. Differential expression analysis was performed using edgeR. Differentially expressed miRNAs between two samples were defined as those with Benjamín-Hochberg false discovery rate (FDR) less than 0.05 (FDR < 0.05) and log2 of fold_change more than one.

### Isoform identification and quantification

To identify the isoforms, the PacBio full-length transcripts were downloaded and mapped to the new lotus reference genome as previously described [55]. Illumina RNA-Seq from petiole, leaf, petal, anther, unpollinated carpel and pollinated carpel were also downloaded to quantify isoform expression. In brief, the high-quality reads were mapped to the reference by HISTA2 v2.1.0, and then the FPKM (fragments per kilobase per million) value of the genes and isoforms were calculated by StringTie v1.3.5 using a combination of Illumina and full-length transcript based annotations. The differentially expressed genes and isoforms were identified by the edgeR package using the same threshold used for miRNA differential expression analysis.

### Prediction of miRNA target genes and isoforms

Comparing with the animal miRNA, most miRNAs and their target mRNAs in the plant have near-perfect sequence complementarity [60]. The miRNAs target isoforms were predicted by TargetFinder [61], with a strict screening criterion. Briefly, FASTA search was applied to find the potential targets, while a position-dependent mispair penalty system was used to score the target sequence after assessment of penalties for mismatches, bulges, gaps (+ 1 per position) and G:U pairs (+ 0.5 per position) [62]. Penalties were doubled if the mismatch, bulge, gap, or G:U pairs occurred at position 2 to 13 relative to the 5′ end of the miRNA. Only one single nucleotide bulge or singe nucleotide gap was allowed while other parameters were set at default. Finally, only predicted targets with scores of three or less were retained.

### Gene ontology, KEGG pathway analyses, and ortholog identification

The GOseq package based on Wallenius non-central hypergeometric distribution [63] was used to identify the significantly enriched GO terms and KEGG pathways for each gene set, including miRNA target genes and the differentially expressed genes. The KEGG pathway annotation was performed using the target gene as queries in GhostKOALA (https://www.kegg.jp/). The orthologs among Arabidopsis (TAIR11), rice (http://rice.plantbiology.msu.edu/, version 7.0) and lotus were identified using the OrthoMCL with an e-value <1e-15 and an inflation parameter of 2.0.

### Co-expression network analysis of transcript isoforms by WCGNA

To determine whether isoform expression is correlated with the tissues, the co-expression networks were constructed based on 15 RNA-seq samples by using WGCNA (v1.0) in R [64]. These 15 samples include five leaf samples, two petioles, two mature anthers, two pollinated carpels, and unpollinated carpels (PRJNA503979 and PRJNA492157). To filter out the silent or constantly expressed isoforms, those with an averaged FPKM > 0.1 and coefficient of variation (C.V.) of FPKM > 2 were retained for the subsequent pipeline of WGCNA. According to Topological Overlap Matrix [65], the transcripts were first clustered hierarchically. The transcripts were assigned to nine co-expression modules using a bottom-up algorithm known as the dynamic hybrid cut method, which was named after randomly assigned colors. Modules were identified under the minimum module size of 600, and the threshold of the module similarity was set at 0.5. The kME of isoform, which measures the correlation between isoforms and modules, was calculated. The top 150 isoforms, which were most correlated with each corresponding module, were defined as hub isoforms according to the WGCNA protocol.

### RT-qPCR analysis of sampled target genes

The cDNA libraries were constructed using 1 μg total RNA from five samples and they were diluted to 100 μL before performing RT-PCR. The miRNA targeted gene-specific primers were designed by using Primer Premier (v.5.0) (Additional file 2: Table S8). The qRT-PCR reactions were performed on QuantStudio 6 Flex (Life Technologies, USA) in a final volume of 10 μg containing 1 μg cDNA. The reaction procedure was initiated at 95 °C for 10 min, followed by 42 cycles of 95 °C for 15 s, 60 °C for 30 s, and 72 °C for 30 s with fluorescence detection. The miRNA targeted gene expressions were analyzed using the 2^-△△CT^ method, with the lotus *β-Actin* gene used as an internal standard.

## Supplementary information

**Additional file 1:.** Figures S1-S19.

**Additional file 2:.** Table S1-S8. (XLS 12087 kb)

## Data Availability

All data analyzed during this study are included in this published article or its supplementary information files. Sequencing raw data used in this study are avaliable in NCBI under accession number PRJNA503979 and PRJNA492157.

## Abbreviations

AS: Alternative Splicing
DEG: Differentially Expressed Gene
DEMTG: Differentially Expressed miRNA Targeted Gene
FDR: False Discovery Rate
FPKM: Fragments Per Kilobase of transcript per Million fragments mapped
GIDDM: Genes have their Isoforms Divided into Different Modules
GO: Gene Ontology
KEEG: Kyoto Encyclopedia of Gene and Genomes
MIRNAs: miRNA genes
TPM: Transcripts per million reads
WGCNA: Weight Gene Co-expression Network Analysis
