# Supplementary material for: Integrative expression network analysis of microRNA and gene isoforms in sacred lotus
Source: BMC Genomics. 2020 Jun 25;21:429. doi: 10.1186/s12864-020-06853-y (PMC7315500; doi:10.1186/s12864-020-06853-y)

Supplement figure

Fig. S1 The distribution of reads along with mature miRNA length

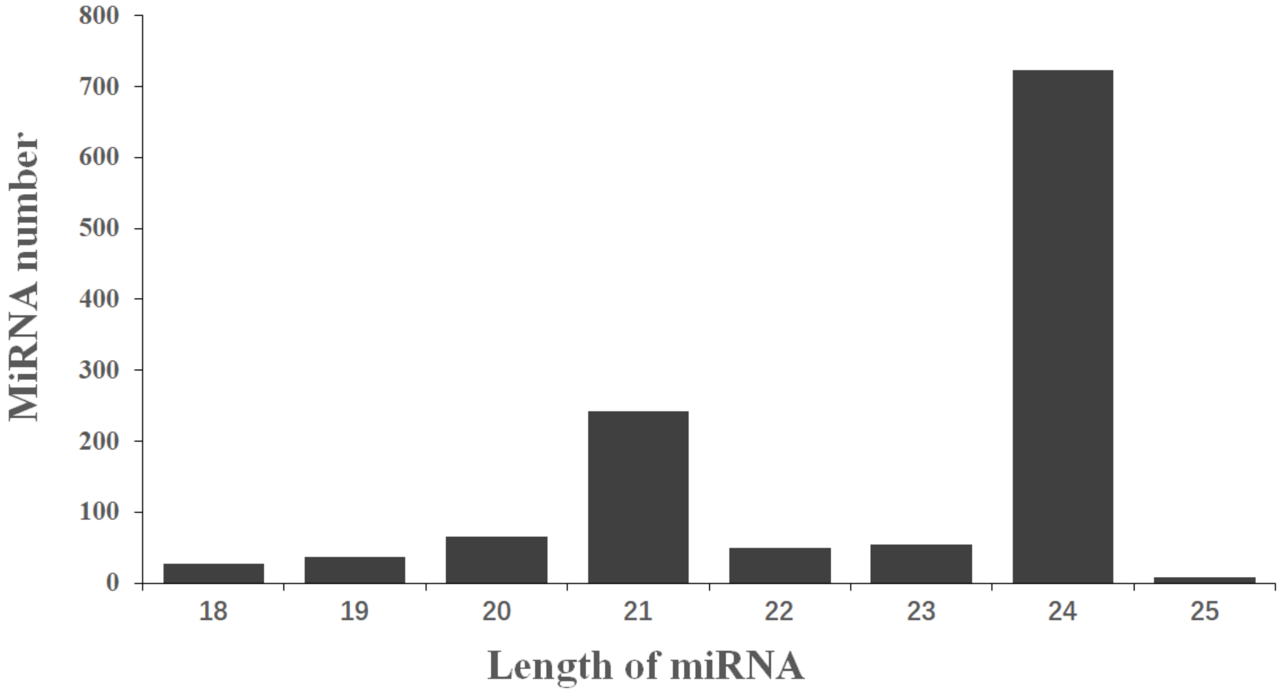

Fig. S2 The presence of the miRNAs nucleotide bias at each position along with the matue miRNAs length

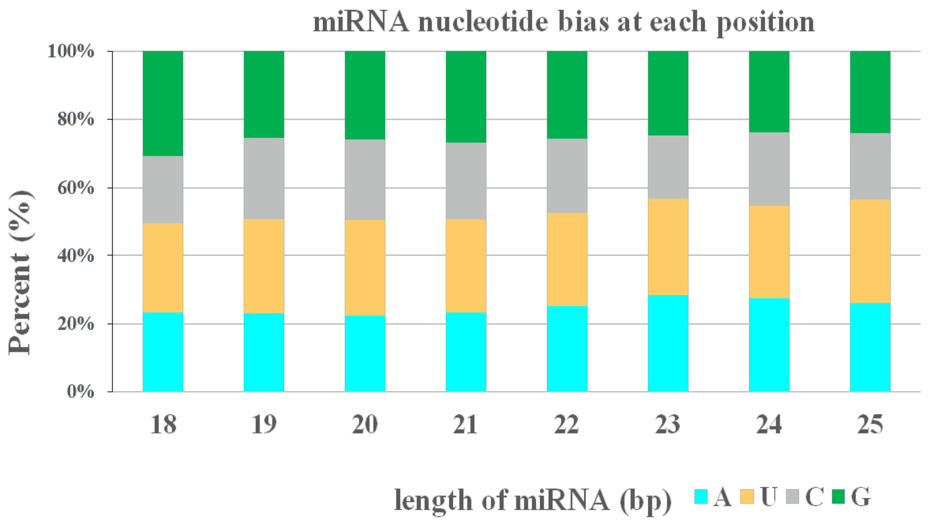

Fig. S3 The presence of the first nucleotide of miRNAs along with the mature miRNA length.

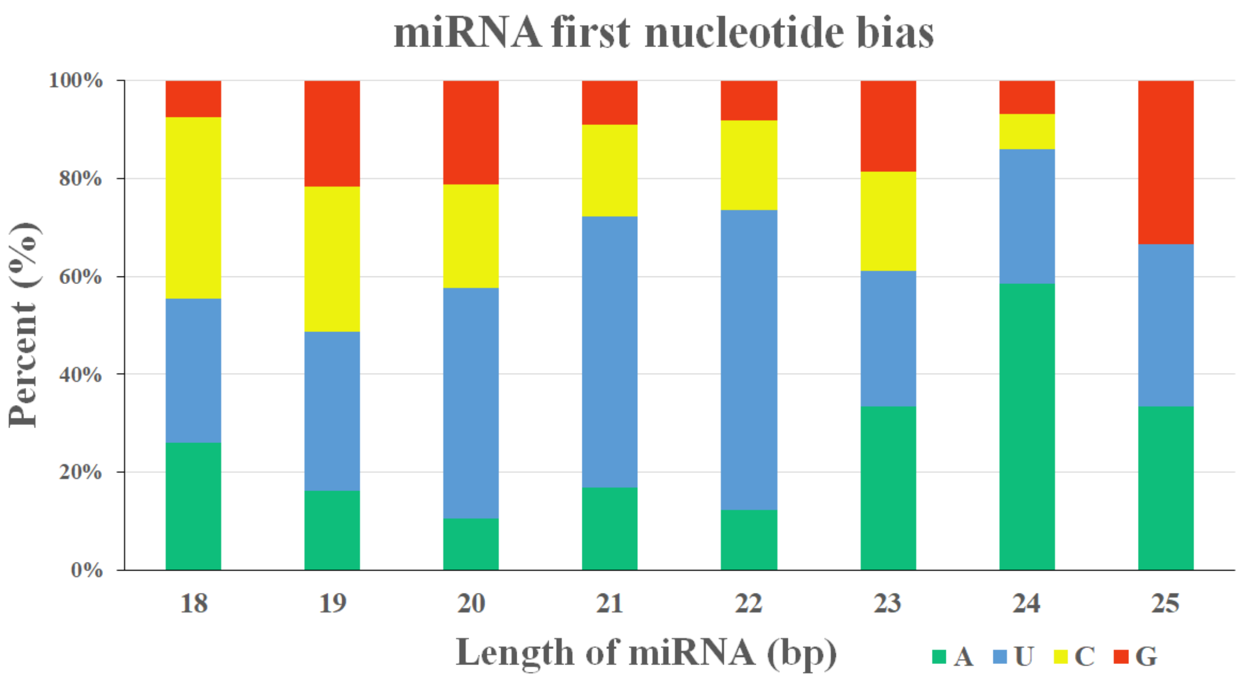

Fig. S4 Pearson correlation coefficients among gene expression profiles generated by RNA-seq analysis of biological replicates.

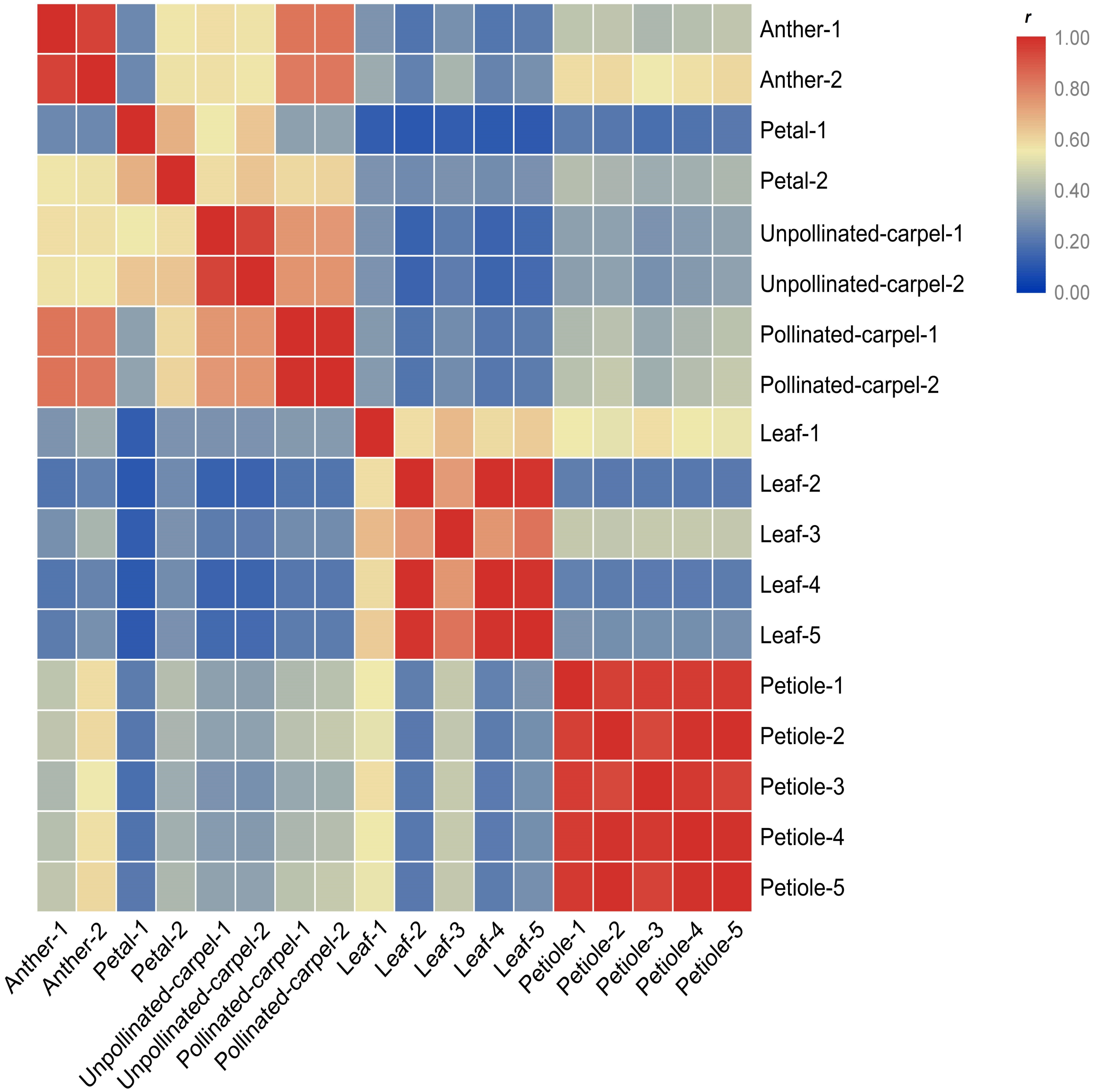

Fig. S5 Expression profile of selected miRNAs and targeted genes. The expression of miRNAs are showed in line graphs. The expression of targeted genes are showed in the bar graph. The correlation coefficient between miRNA and targeted gene is showed. Gray : RT-PCR result; red: RNA-seq result. (a-f) “nnu-miR394” and its corresponding targeted gene. (e-h) “miR159-3p” and its corresponding targeted gene.

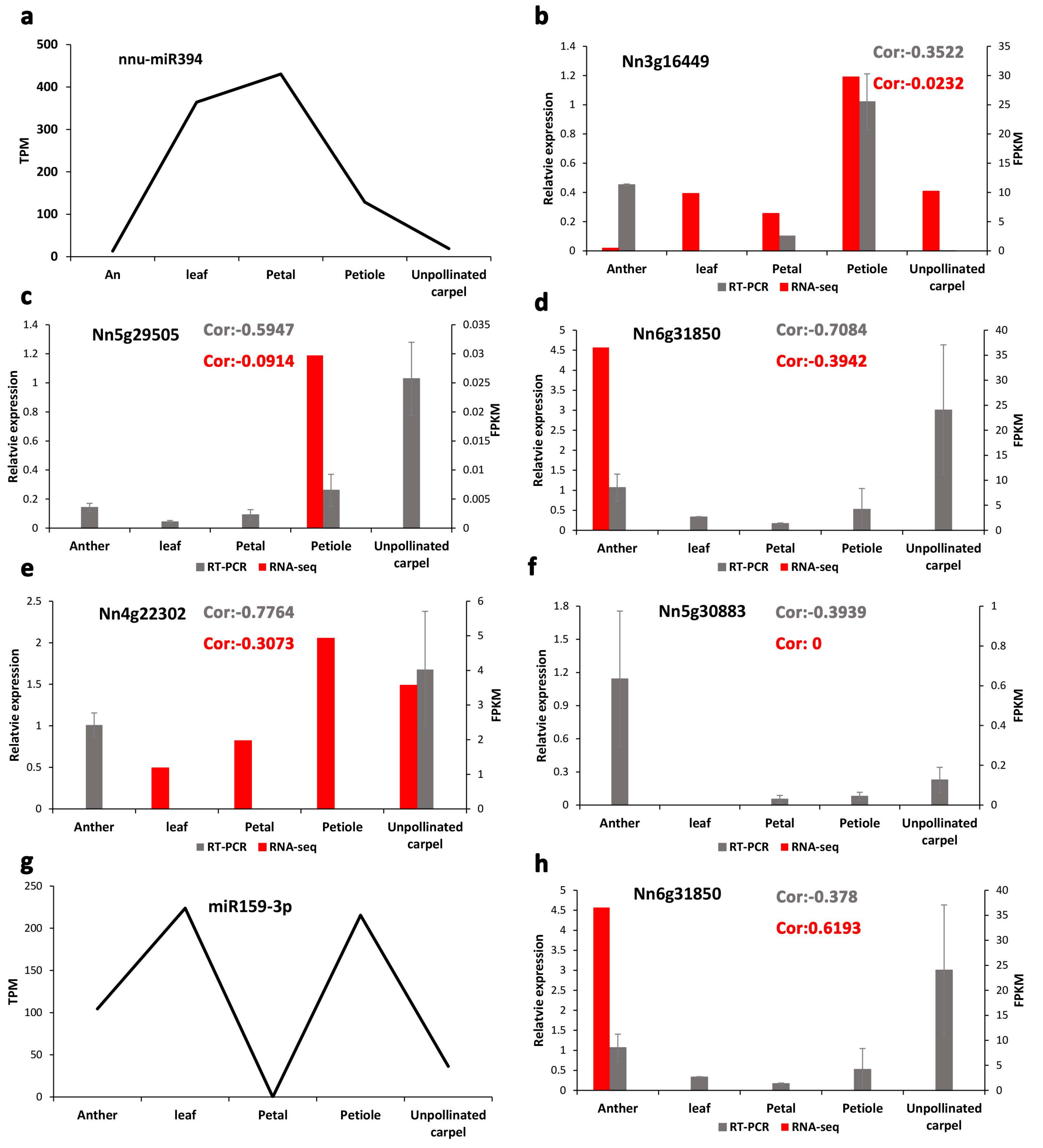

Fig. S6 Characterization of identified miRNAs target loci of lotus. a) The distribution of miRNAs target loci in genes. b) The distribution of miRNAs target loci in isoforms

a

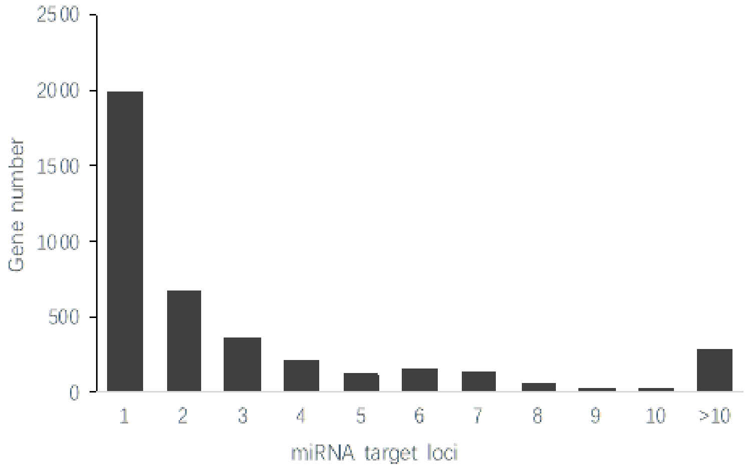

b

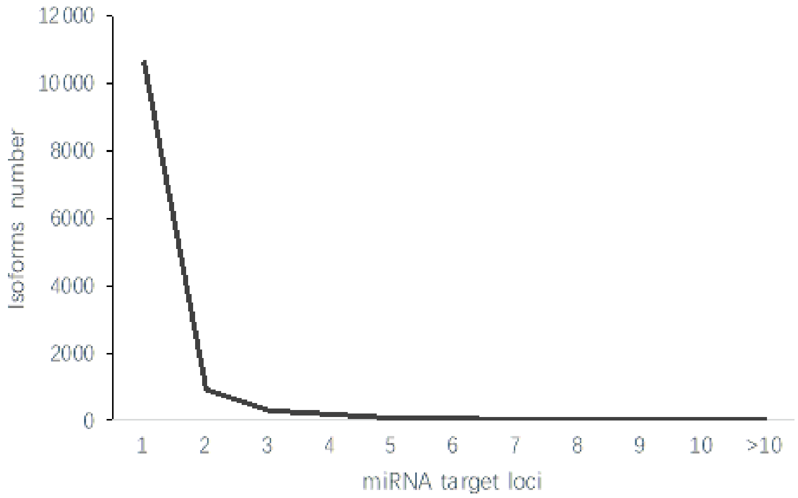

Fig. S 7 The target analysis of miRNA and isoforms. The upper part show that the blue box-line is the construct of isoforms and the down part was the target loci of gene's different isoforms.

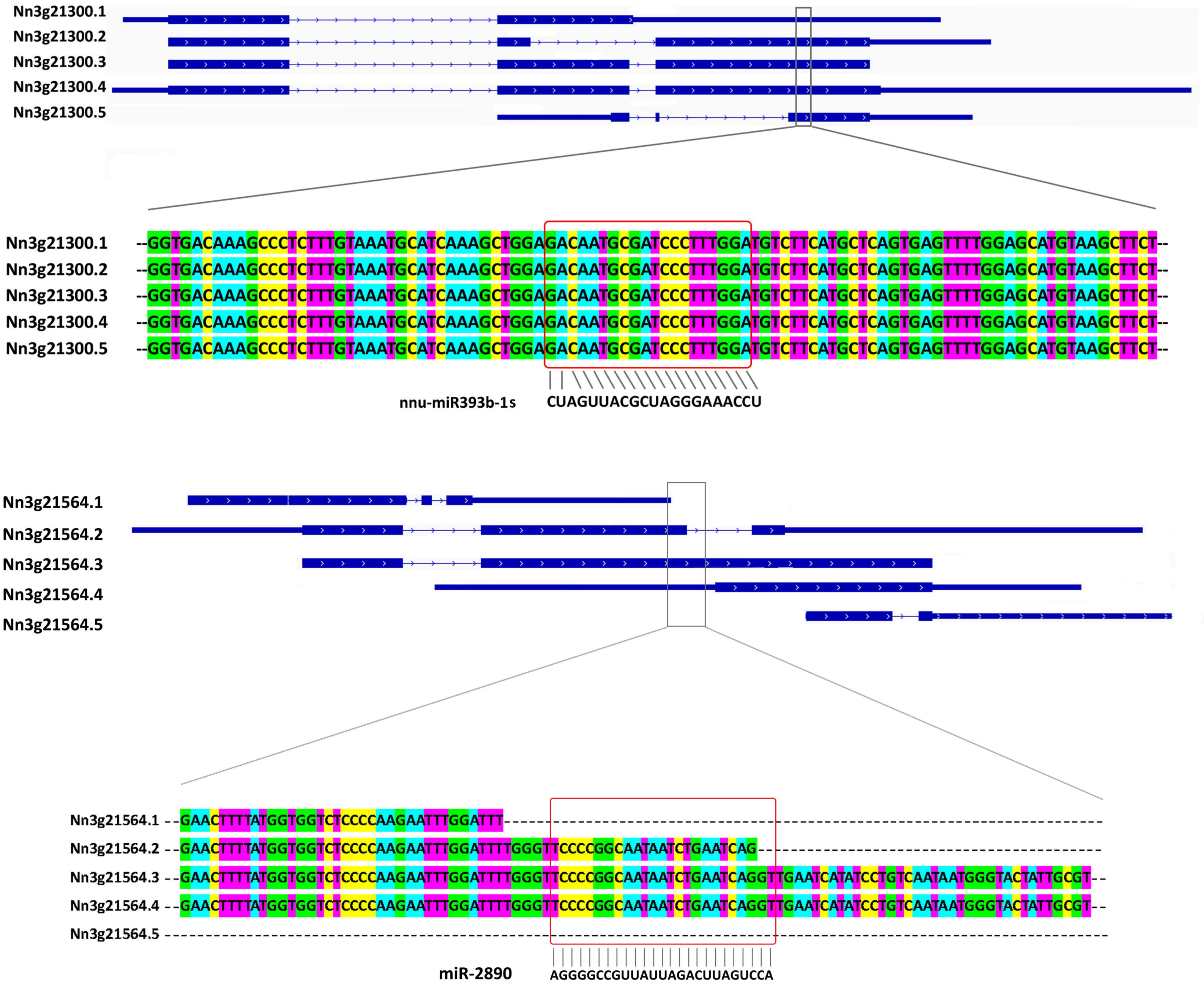

Fig. S8 The expression analysis of miRNA targeted isoforms and non-miRNA targeted isoforms from same genes in lotus six samples.

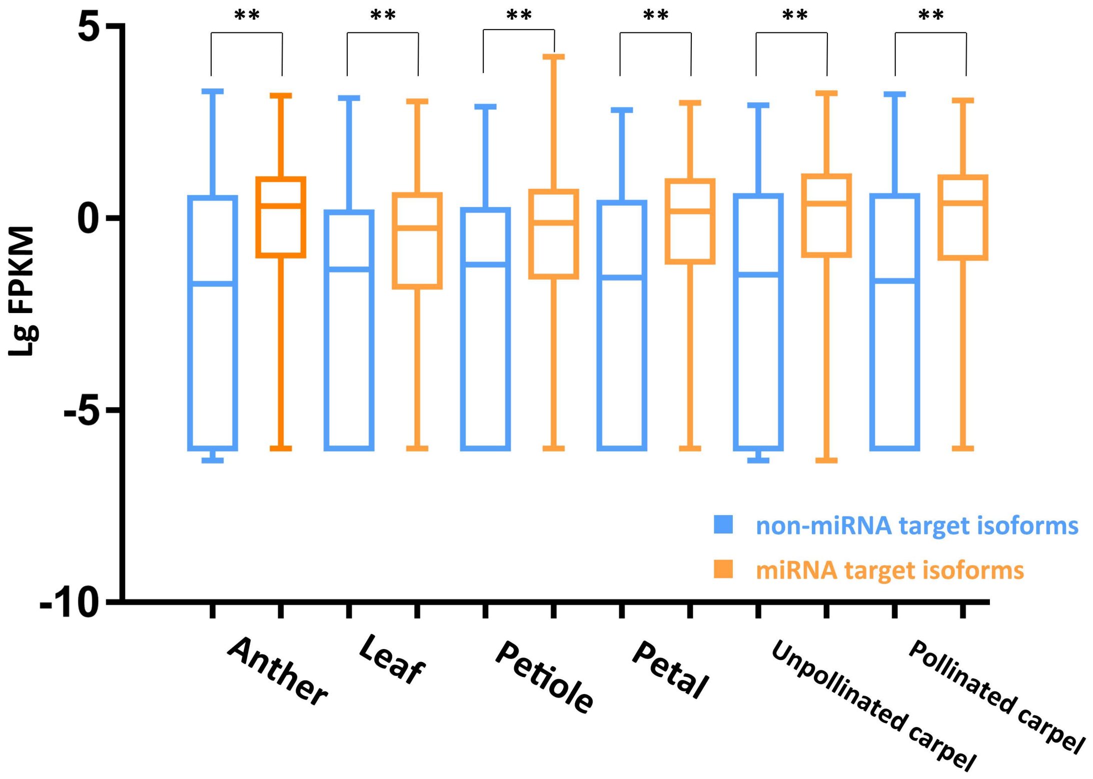

Fig. S9 The GO and its enrichment analysis of all miRNAs target genes

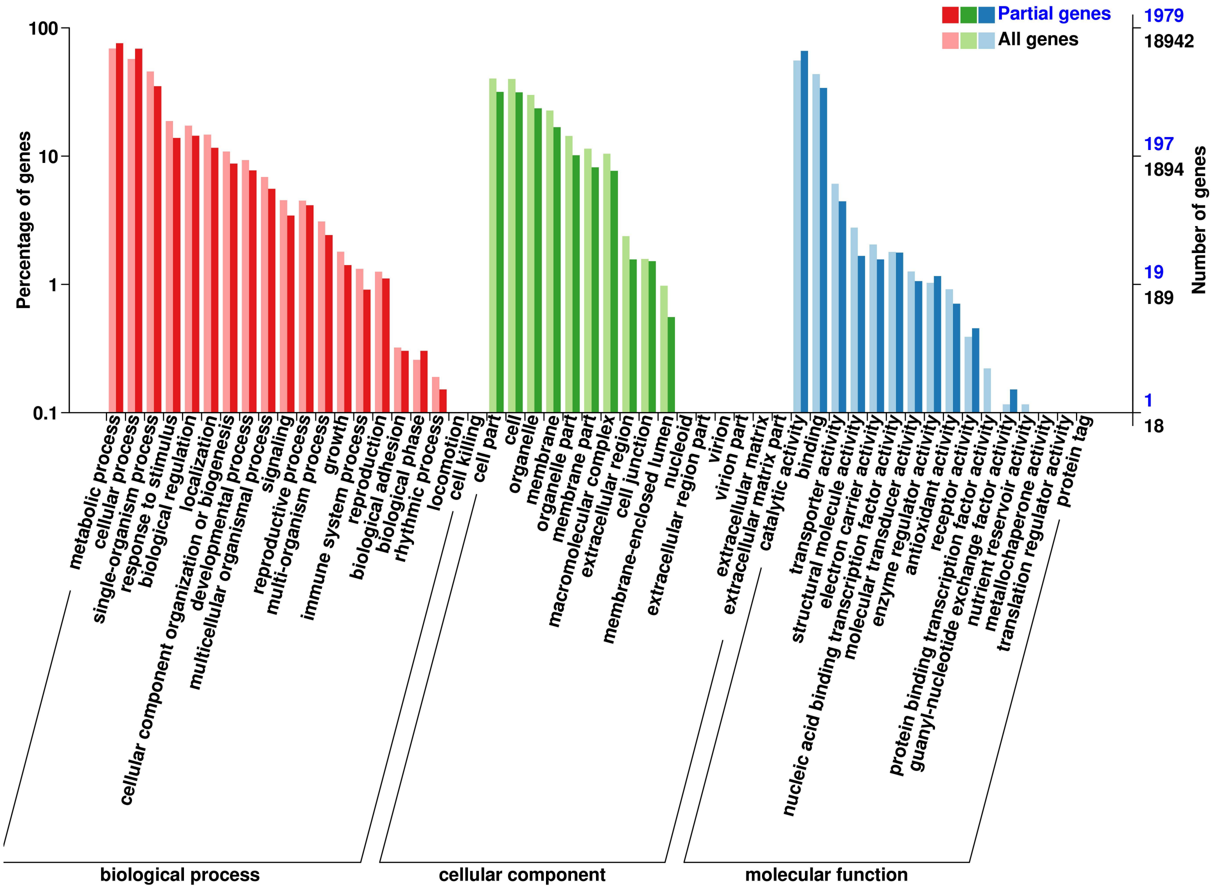

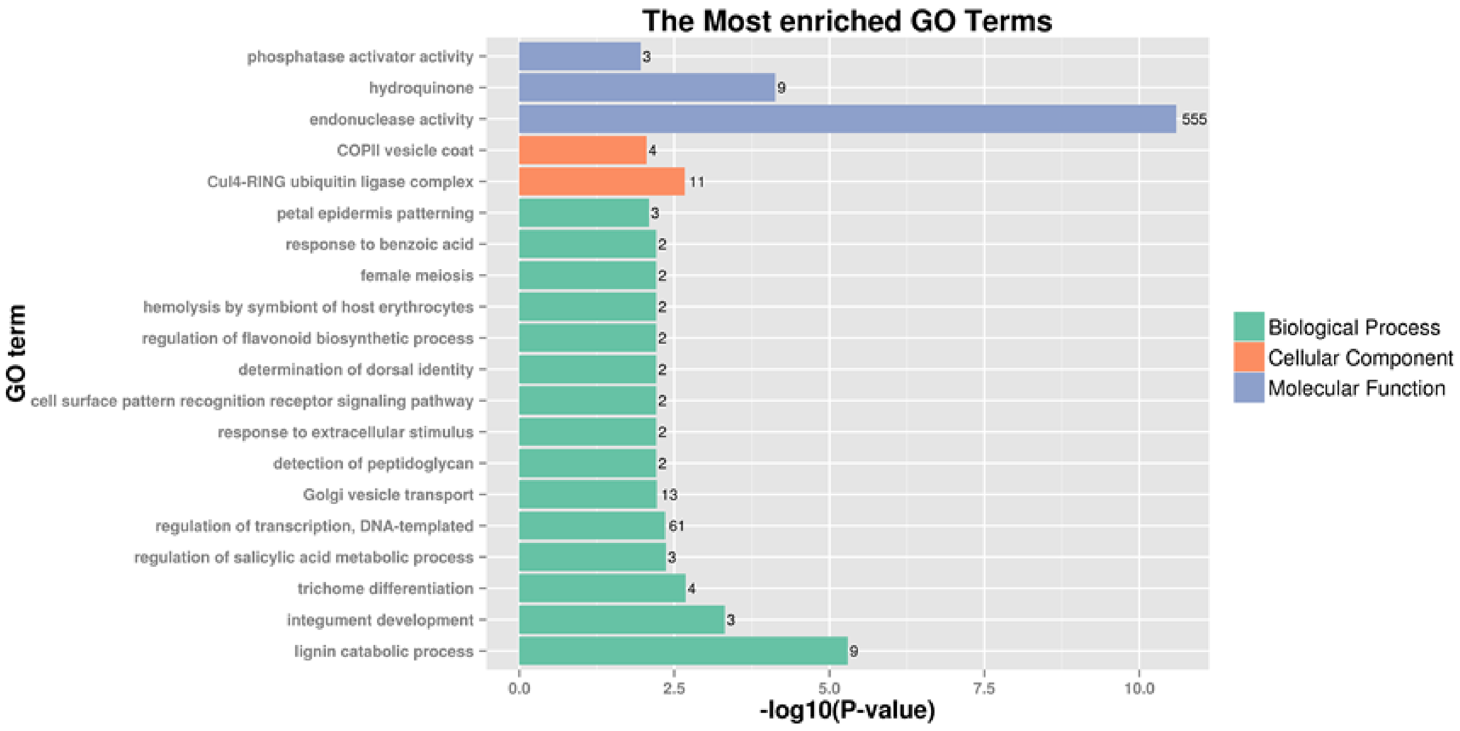

Fig. S10 The GO enrichment analysis in 6 samples. a) anther; b) leaf; c)petal; d) petiole; e) unpollinated carpel ; f) pollinated carpel.

Anther

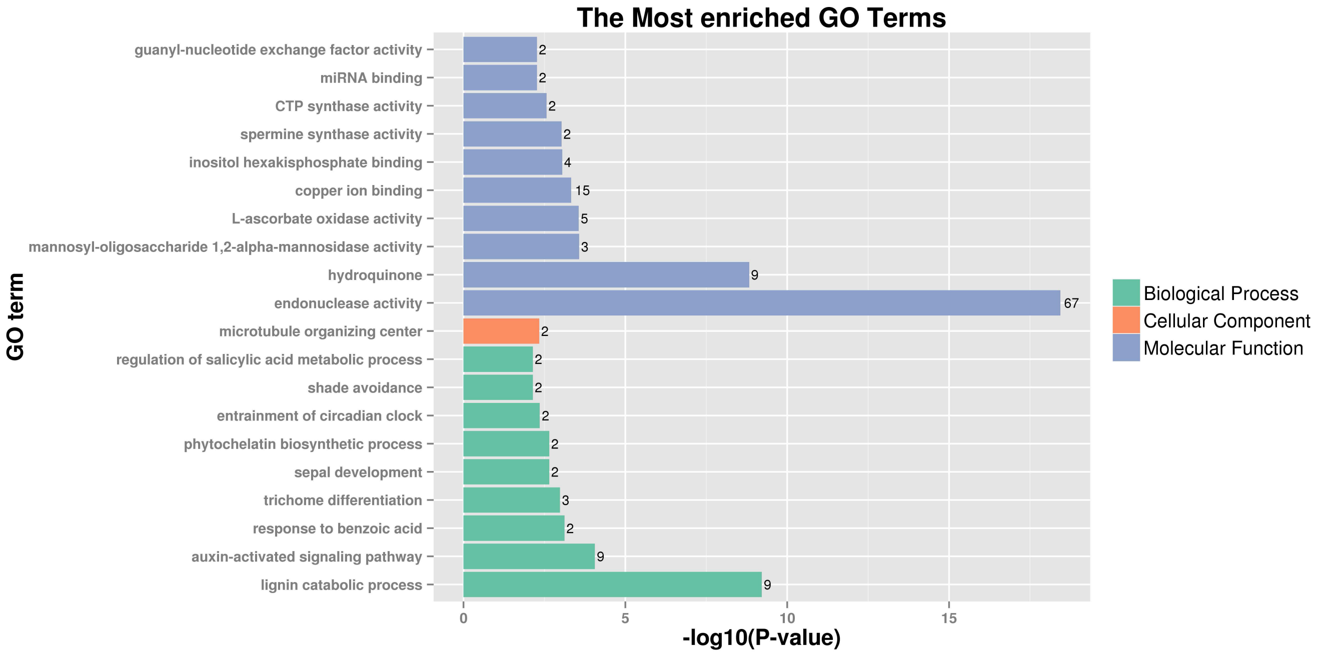

Leaf

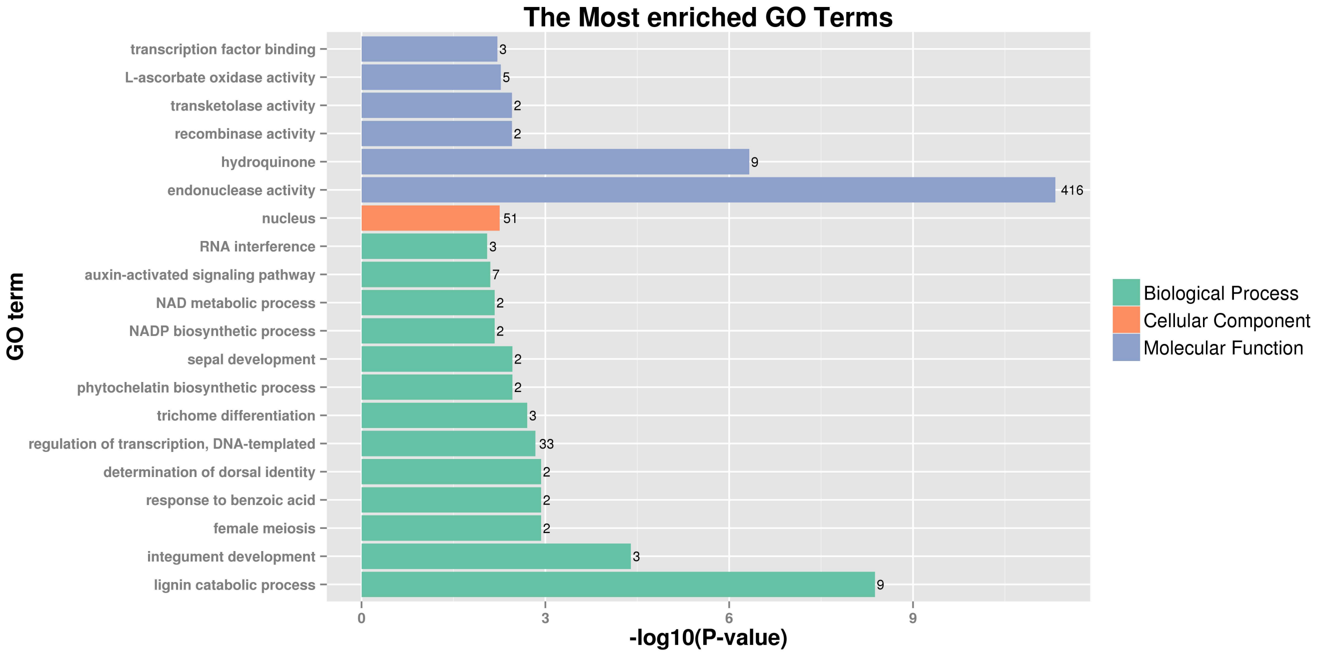

Petal

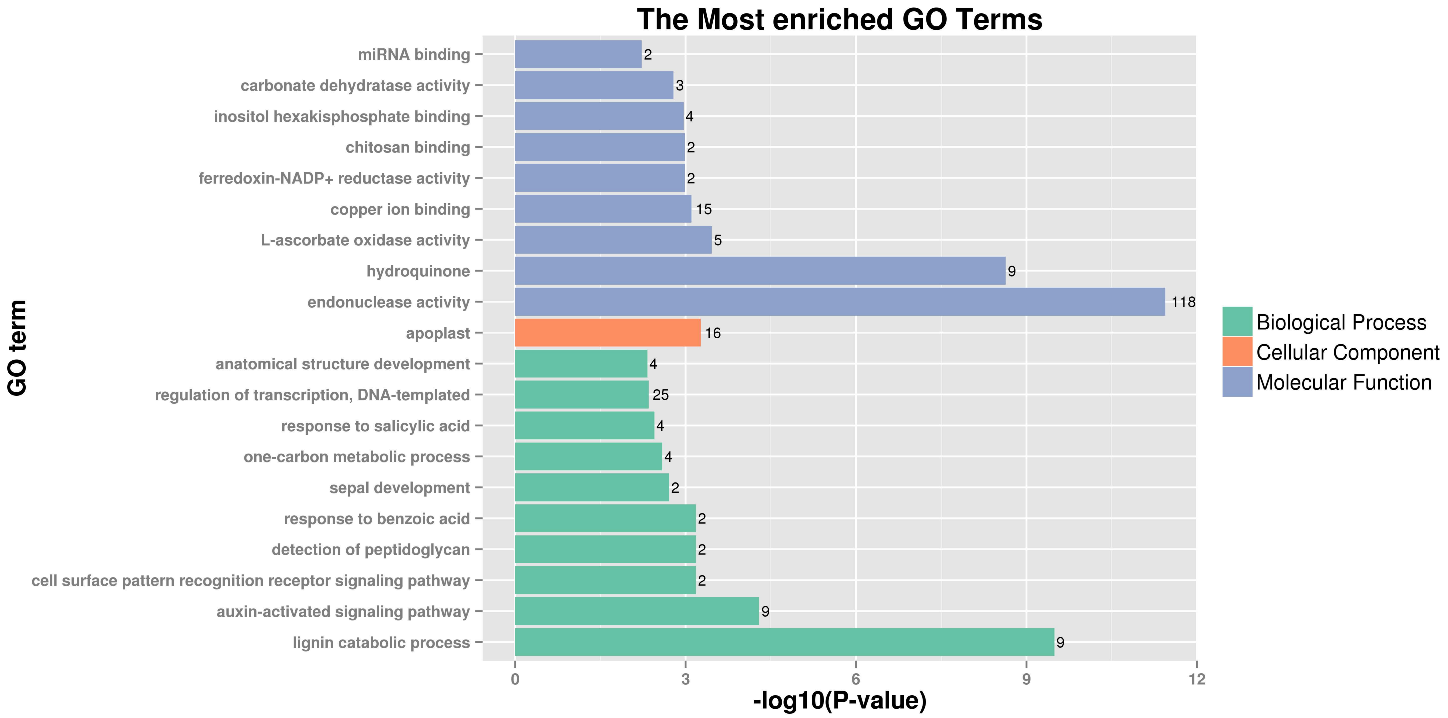

Petiole

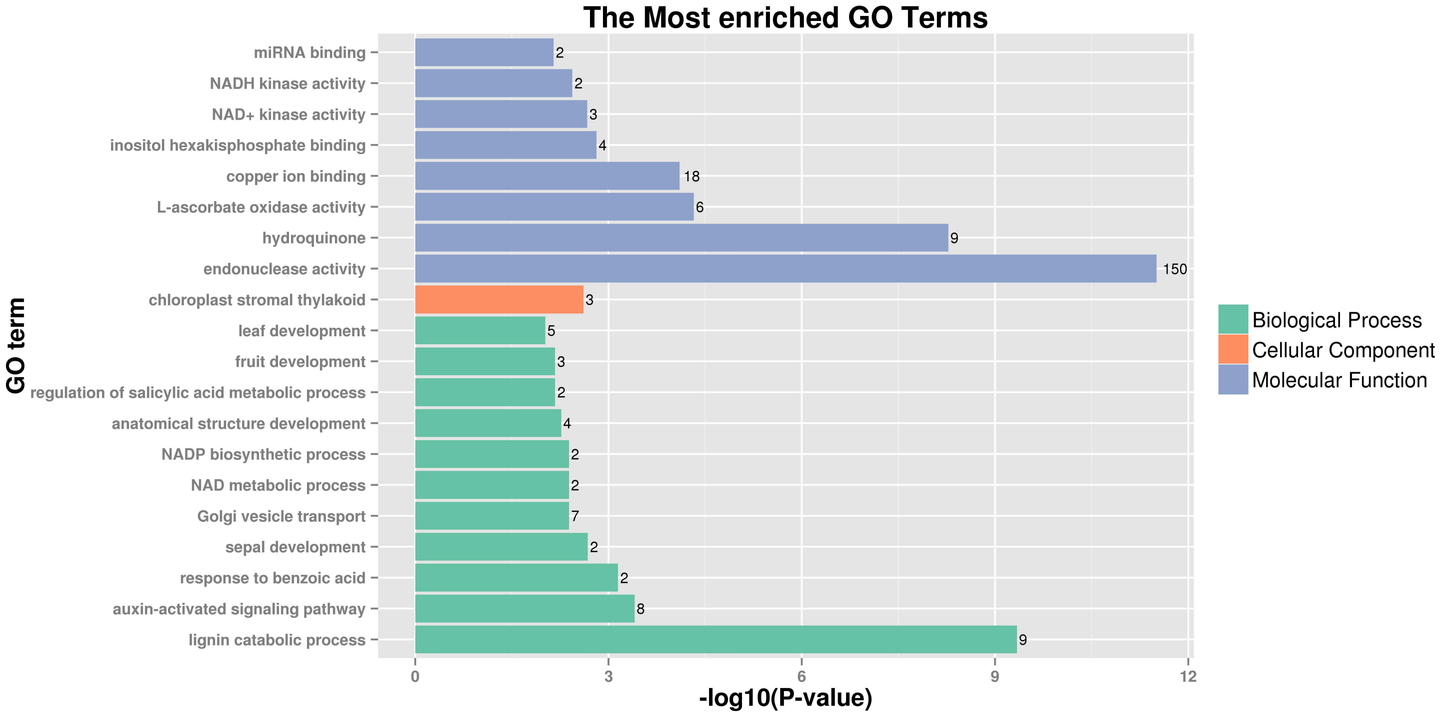

Unpollinated carpel

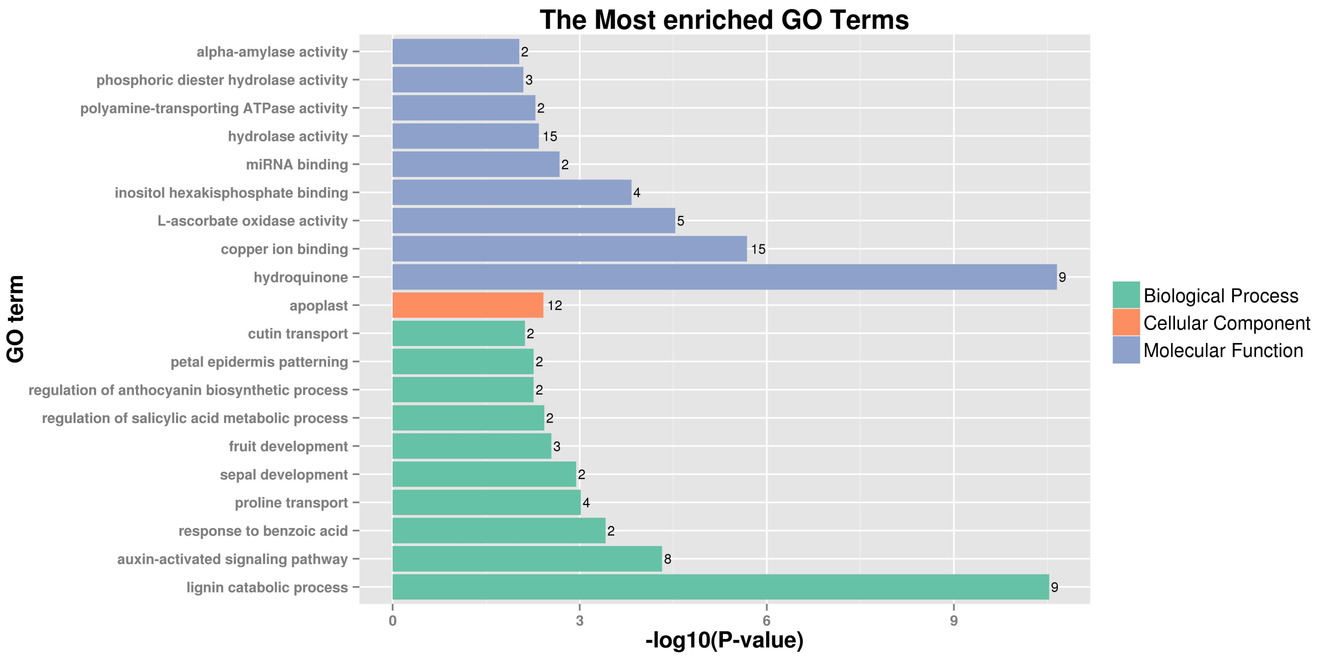

Pollinated carpel

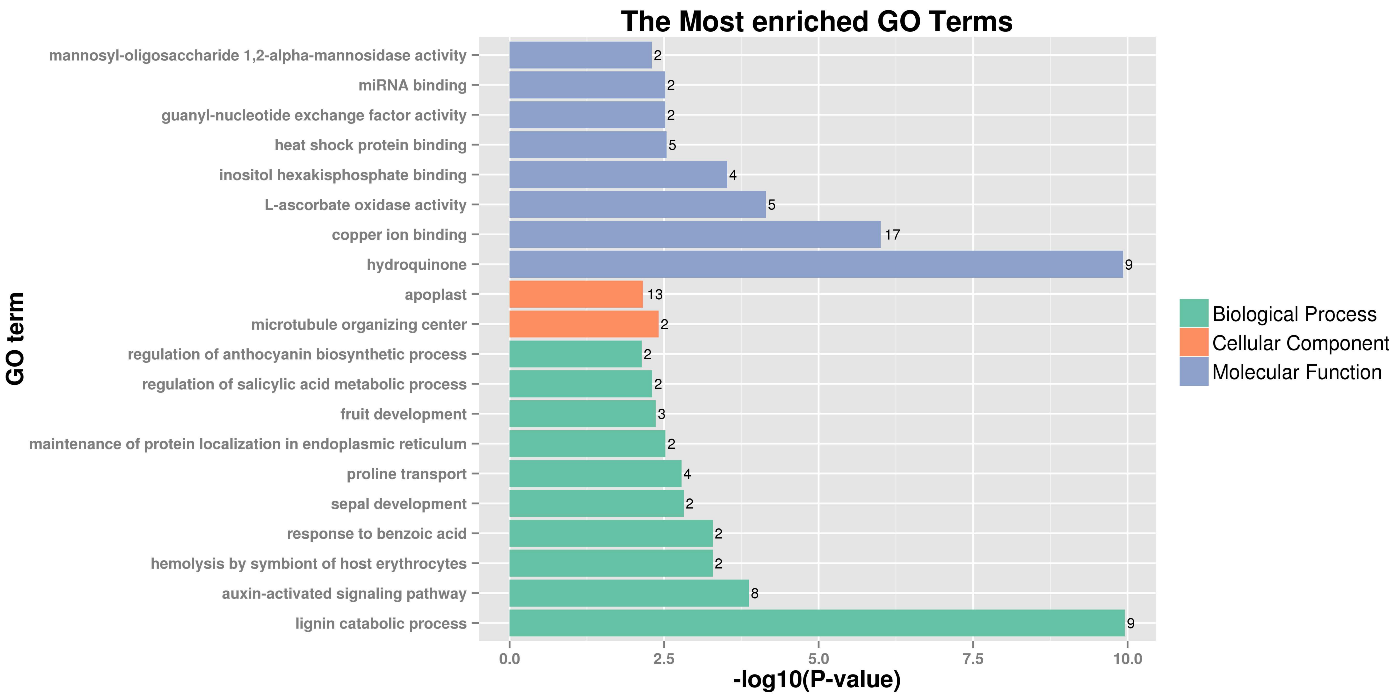

Fig. S11 Heatmap of filtered isoforms in 6 samples. The expression level is displayed with different color based on log2 (FPKM).

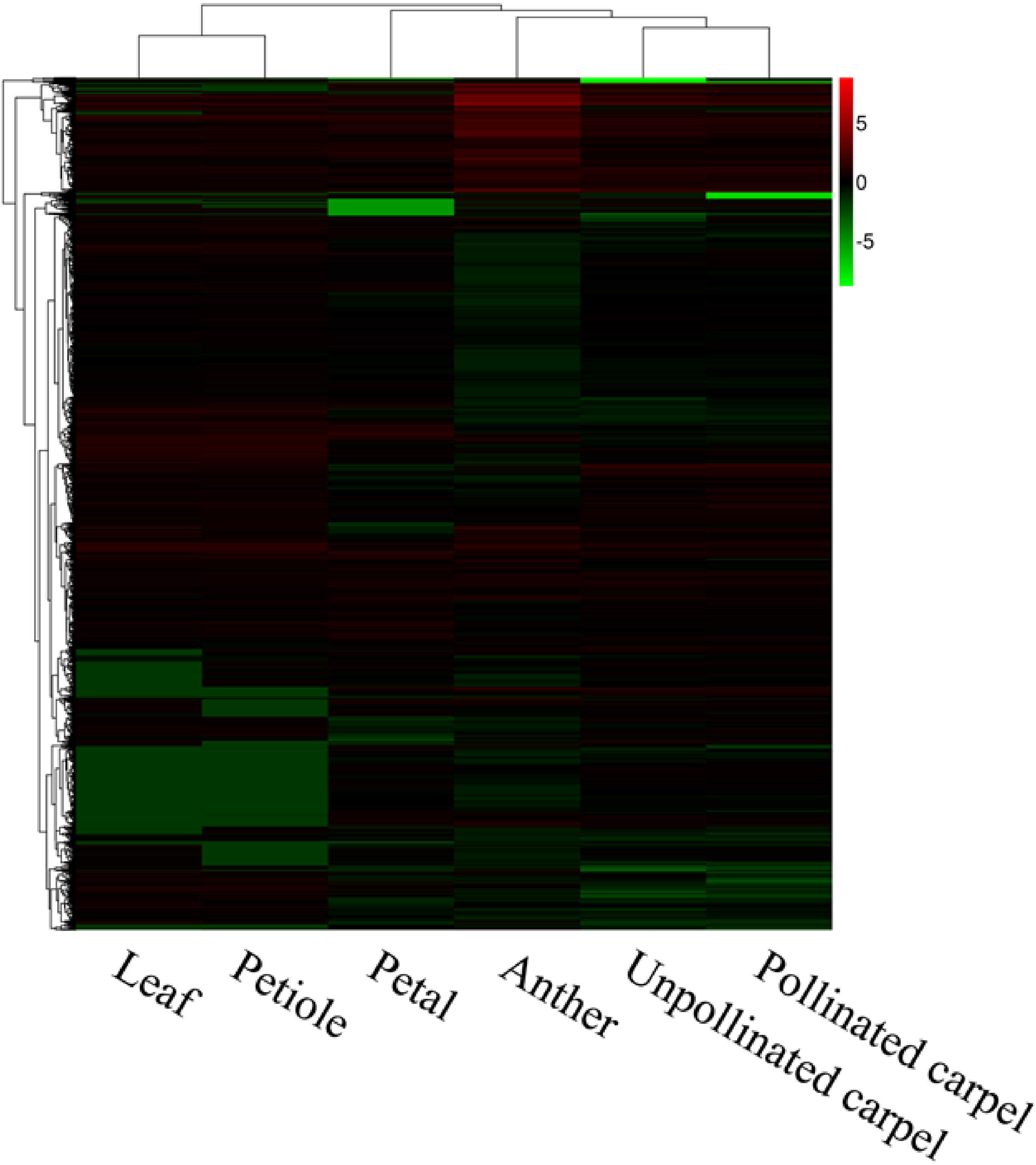

Fig. S12 The correlation network of hub isoforms in each module.

Black

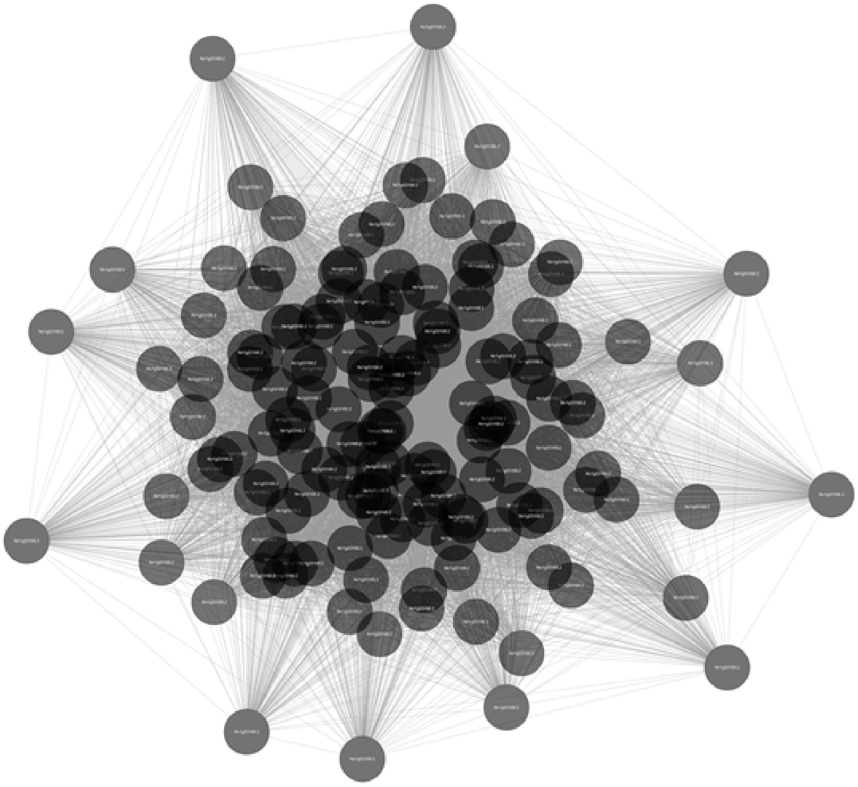

Brown

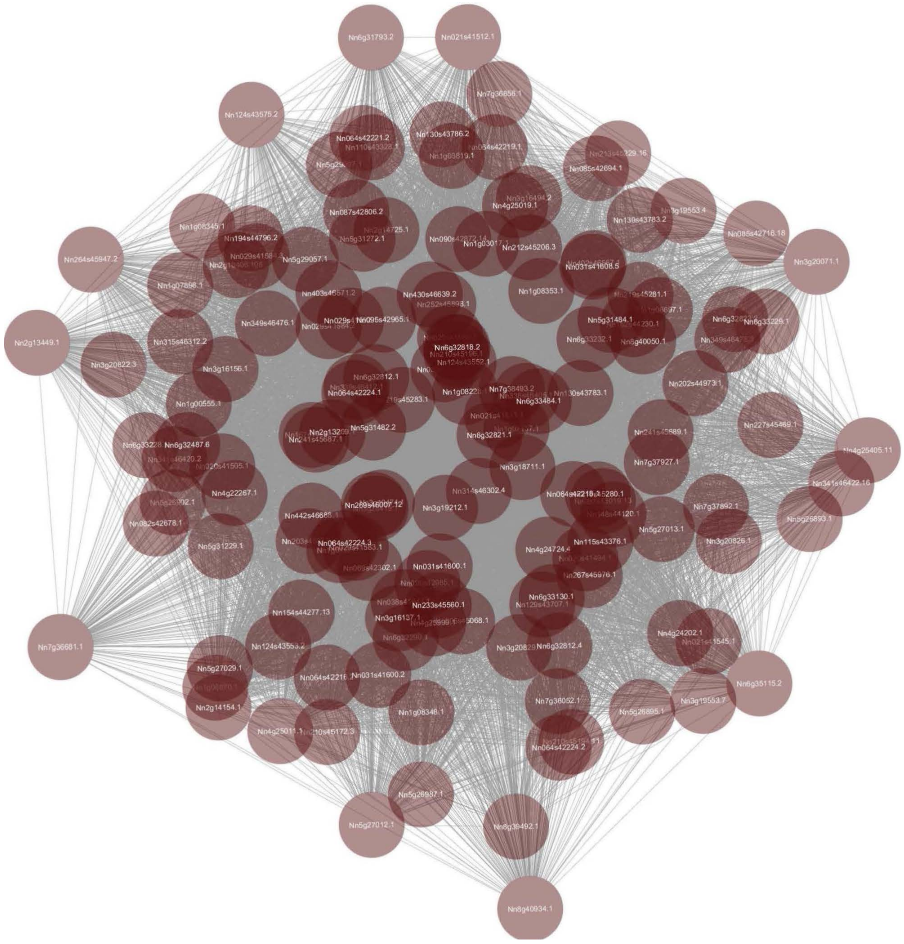

cyan

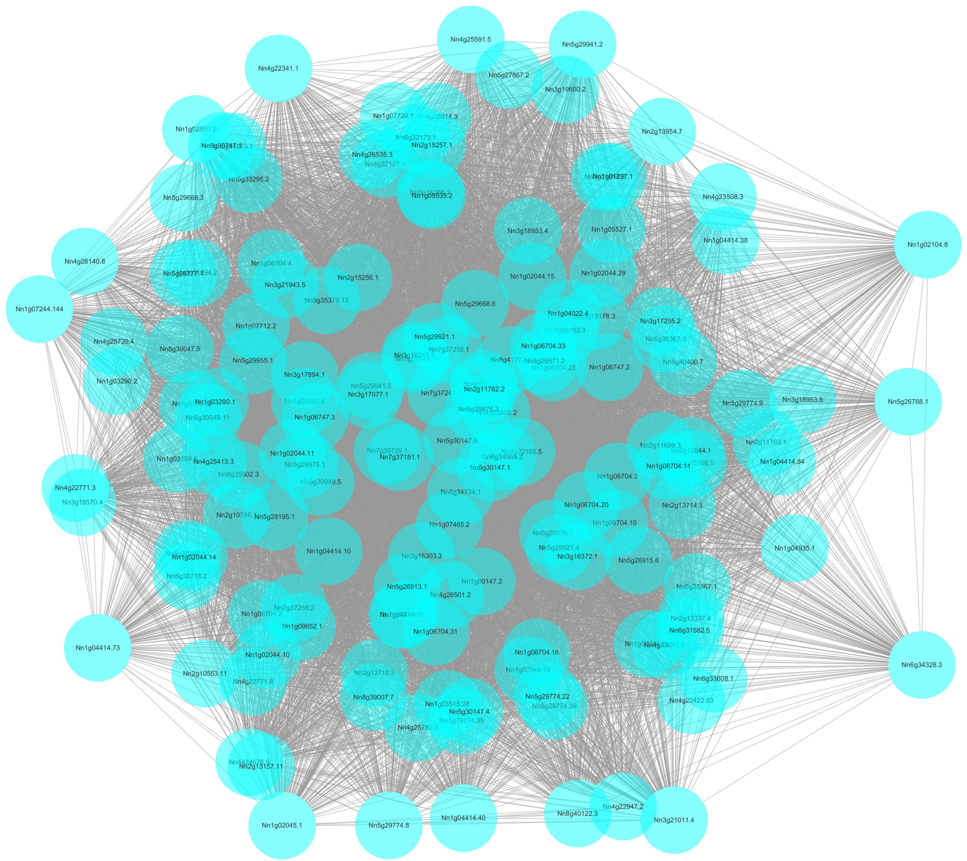

Nn4g25591.5
Nn5g29941.2
Nn4g22341.1
Nn6g27867.2
Nn3g19600.2
Nn2g13954.7
Nn4g23508.3
Nn1g02104.8
Nn5g29668.3
Nn4g26140.8
Nn1g07244.144
Nn5g26788.1
Nn3g18953.8
Nn4g22771.3
Nn3g18570.4
Nn1g04935.1
Nn1g04414.73
Nn6g34328.3
Nn1g02045.1
Nn5g29774.8
Nn1g04414.40
Nn8g40122.3
Nn4g22947.2
Nn3g21011.4

green

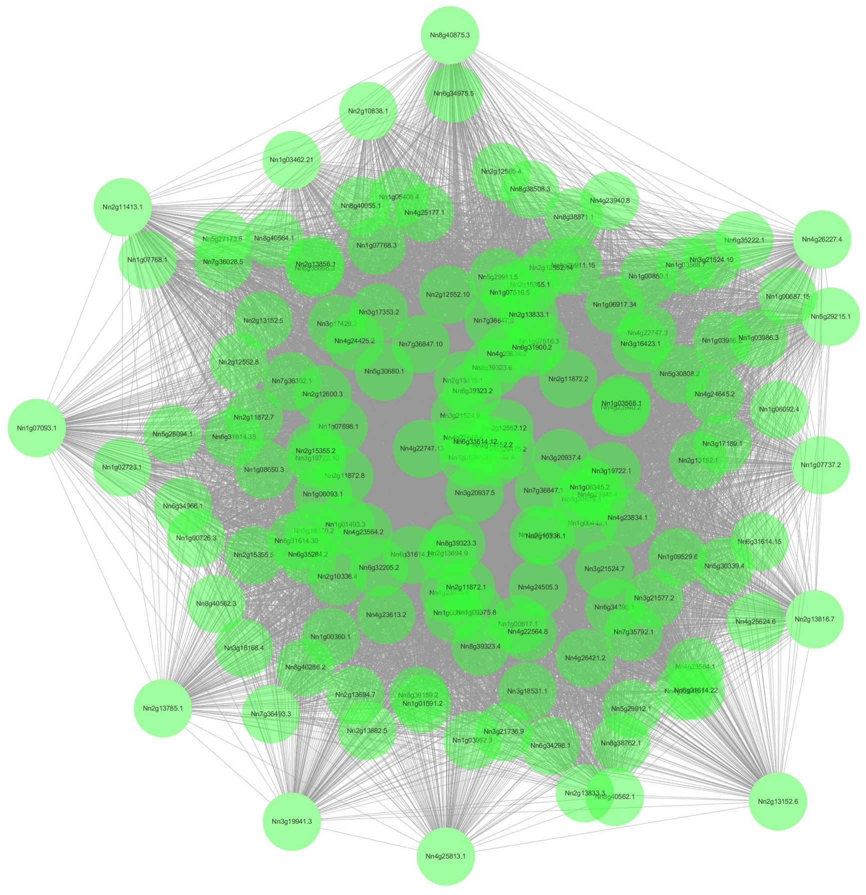

Nn8g40875.3
Nn6g34975.5
Nn2g10838.1
Nn1g03462.21
Nn2g11413.1
Nn4g26227.4
Nn5g29215.1
Nn1g07093.1
Nn1g02723.1
Nn1g07737.2
Nn2g13816.7
Nn2g13785.1
Nn3g19941.3
Nn4g25813.1
Nn2g13152.6

greenyellow

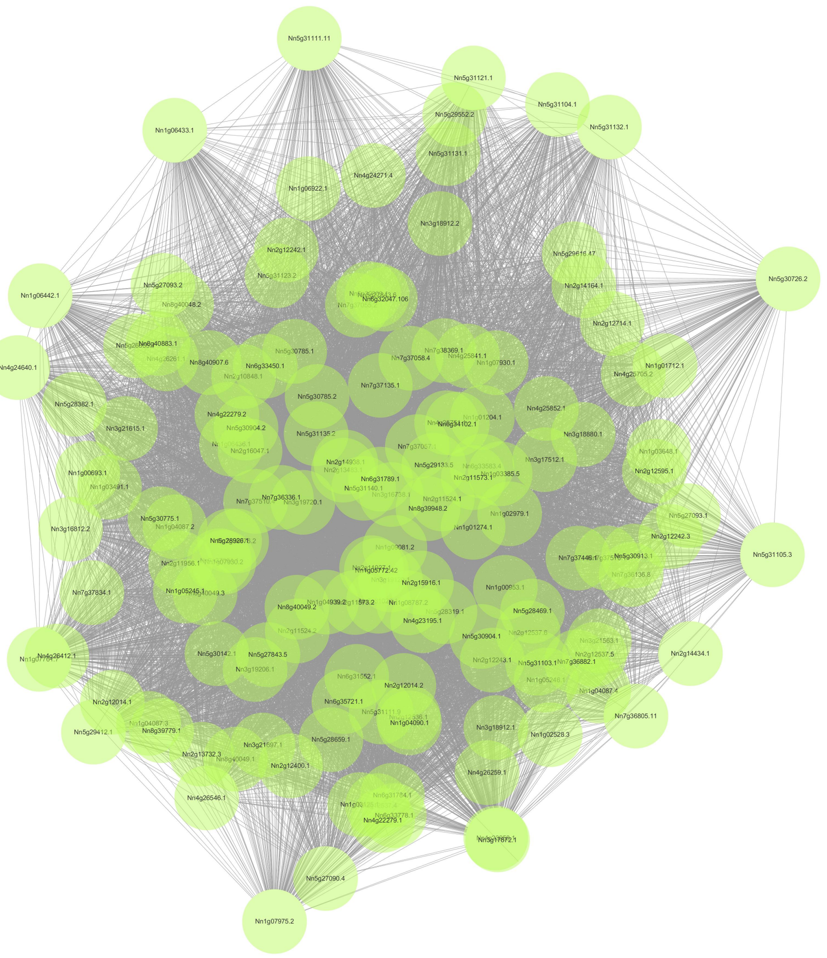

megenta

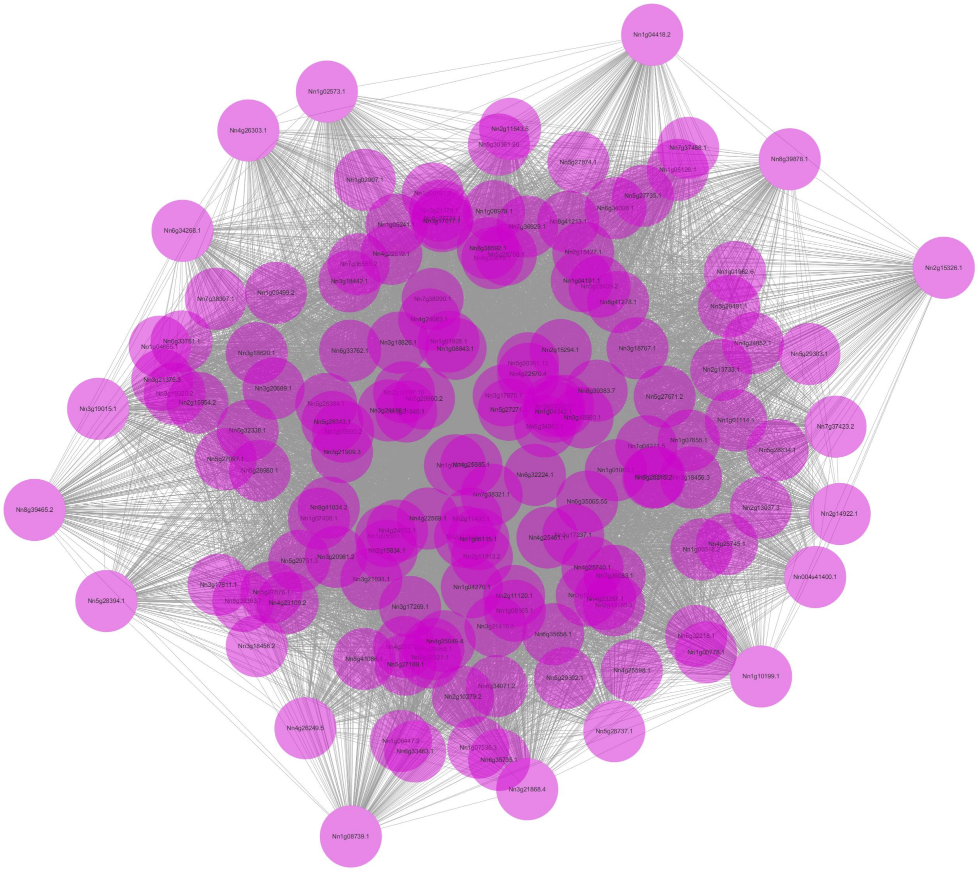

midnightblue

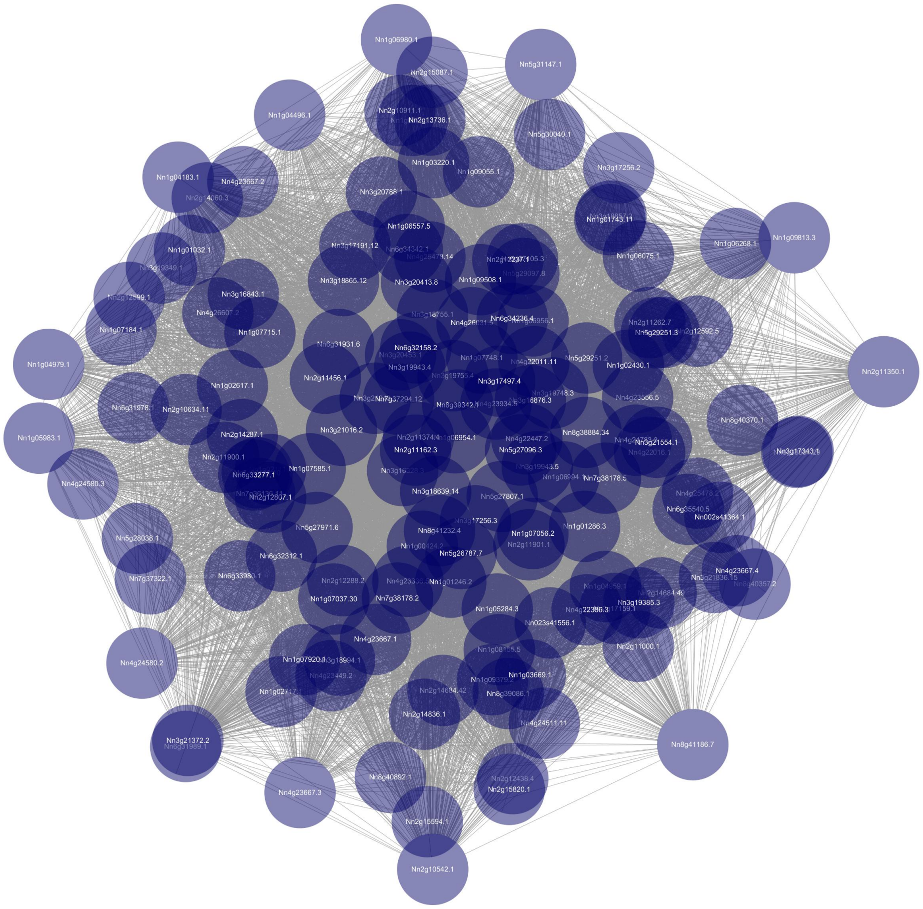

pink

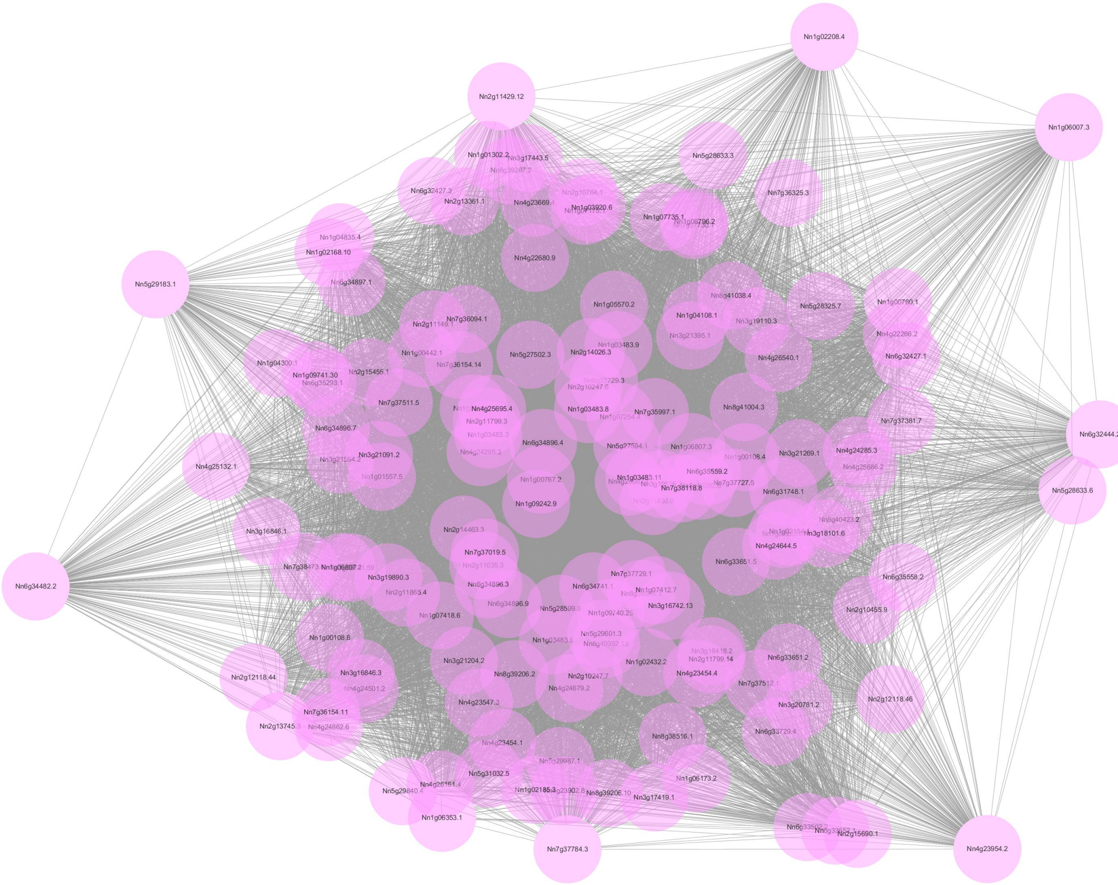

red

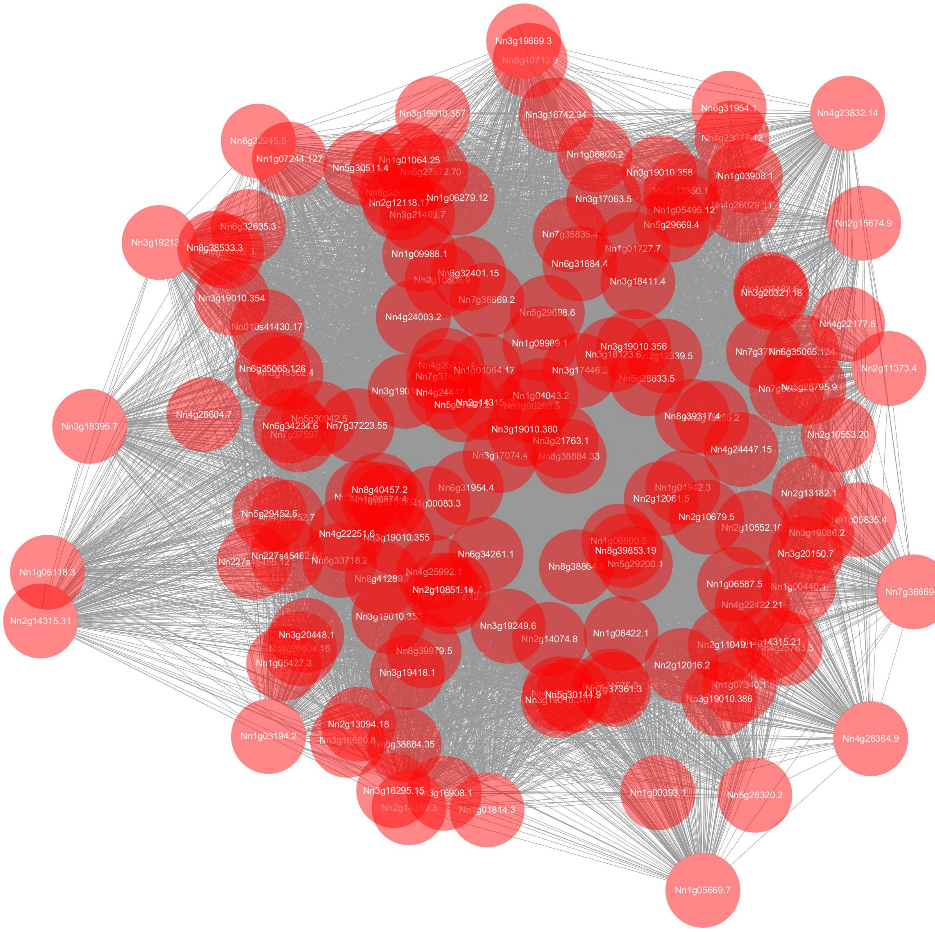

Nn3g19669.3
Nn3g19010.357
Nn3g16742.34
Nn6g31954.1
Nn4g23832.14
Nn1g07244.127
Nn1g01064.25
Nn5g30511.4
Nn1g06800.2
Nn3g19010.358
Nn1g03908.1
Nn2g12118.1
Nn1g06279.12
Nn3g17063.5
Nn1g05495.12
Nn2g15674.9
Nn6g32635.3
Nn8g38533.3
Nn5g29669.4
Nn1g09988.1
Nn6g31684.4
Nn3g18411.4
Nn8g32401.15
Nn3g20321.18
Nn3g19010.354
Nn7g36669.2
Nn5g29698.6
Nn4g22177.6
Nn4g24003.2
Nn1g09989.1
Nn3g19010.356
Nn2g11373.4
Nn6g35065.126
Nn3g17446.3
Nn5g28633.5
Nn3g18395.7
Nn4g26604.7
Nn1g04043.2
Nn6g34234.6
Nn7g37223.55
Nn3g19010.380
Nn8g39317.4
Nn3g21763.1
Nn4g24447.15
Nn2g10553.20
Nn3g17074.4
Nn8g38884.33
Nn6g31954.4
Nn8g40457.2
Nn2g13182.1
Nn2g12061.5
Nn5g29452.5
Nn2g10679.5
Nn4g22251.6
Nn3g19010.355
Nn6g34261.1
Nn8g39853.19
Nn5g29200.1
Nn3g20150.7
Nn1g06118.3
Nn6g33718.2
Nn1g06587.5
Nn7g36669.6
Nn2g10851.14
Nn2g14315.31
Nn3g19249.6
Nn4g22422.21
Nn2g14074.8
Nn1g06422.1
Nn3g20448.1
Nn8g39979.5
Nn2g12016.2
Nn3g19418.1
Nn5g30144.9
Nn3g19010.386
Nn4g26364.9
Nn1g03194.2
Nn2g13094.18
Nn3g16295.15
Nn1g00393.1
Nn5g28320.2
Nn1g01814.3
Nn1g05669.7

yellow

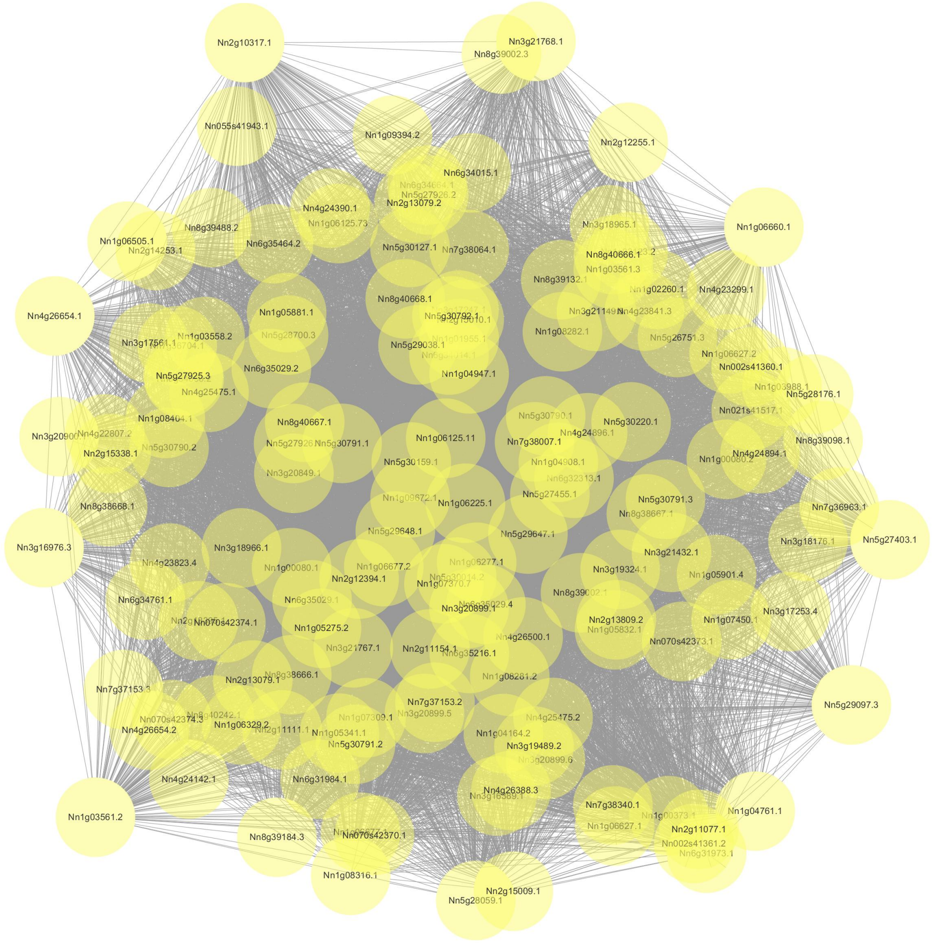

Nn2g10317.1
Nn3g21768.1
Nn8g39002.3
Nn055s41943.1
Nn1g09394.2
Nn2g12255.1
Nn6g34015.1
Nn4g24390.1
Nn2g13079.2
Nn8g39488.2
Nn1g06505.1
Nn2g14253.1
Nn6g35464.2
Nn5g30127.1
Nn7g38064.1
Nn3g18965.1
Nn1g06660.1
Nn8g40666.1
Nn1g03561.3
Nn8g39132.1
Nn4g23299.1
Nn4g26654.1
Nn1g05881.1
Nn8g40668.1
Nn5g30792.1
Nn4g23841.3
Nn3g17561.1
Nn1g03558.2
Nn5g29038.1
Nn1g08282.1
Nn5g26751.3
Nn1g06627.2
Nn002s41360.1
Nn6g35029.2
Nn5g27925.3
Nn1g04947.1
Nn4g25475.1
Nn5g28176.1
Nn1g08404.1
Nn021s41517.1
Nn5g30790.1
Nn5g30220.1
Nn8g40667.1
Nn8g39098.1
Nn5g30791.1
Nn1g06125.11
Nn7g38007.1
Nn2g15338.1
Nn5g30790.2
Nn4g24894.1
Nn3g20849.1
Nn5g30159.1
Nn5g27455.1
Nn1g06225.1
Nn5g30791.3
Nn7g36963.1
Nn8g38668.1
Nn5g29548.1
Nn5g29647.1
Nn3g16976.3
Nn3g18176.1
Nn5g27403.1
Nn3g21432.1
Nn4g23823.4
Nn3g18966.1
Nn1g00080.1
Nn1g06677.2
Nn3g19324.1
Nn1g05901.4
Nn2g12394.1
Nn6g34761.1
Nn070s42374.1
Nn2g13809.2
Nn1g05832.1
Nn3g17253.4
Nn1g07450.1
Nn3g20899.1
Nn1g05275.2
Nn070s42373.1
Nn4g26500.1
Nn3g21767.1
Nn2g11154.1
Nn7g37153.3
Nn2g13079.1
Nn5g29097.3
Nn7g37153.2
Nn070s42374.2
Nn4g26654.2
Nn1g06329.2
Nn1g05341.1
Nn5g30791.2
Nn3g19489.2
Nn4g25475.2
Nn4g24142.1
Nn6g31984.1
Nn4g26388.3
Nn7g38340.1
Nn1g04761.1
Nn1g03561.2
Nn8g39184.3
Nn070s42370.1
Nn2g11077.1
Nn002s41361.2
Nn1g08316.1
Nn2g15009.1
Nn5g28059.1

Fig. S13 The correlation analysis between different modules.

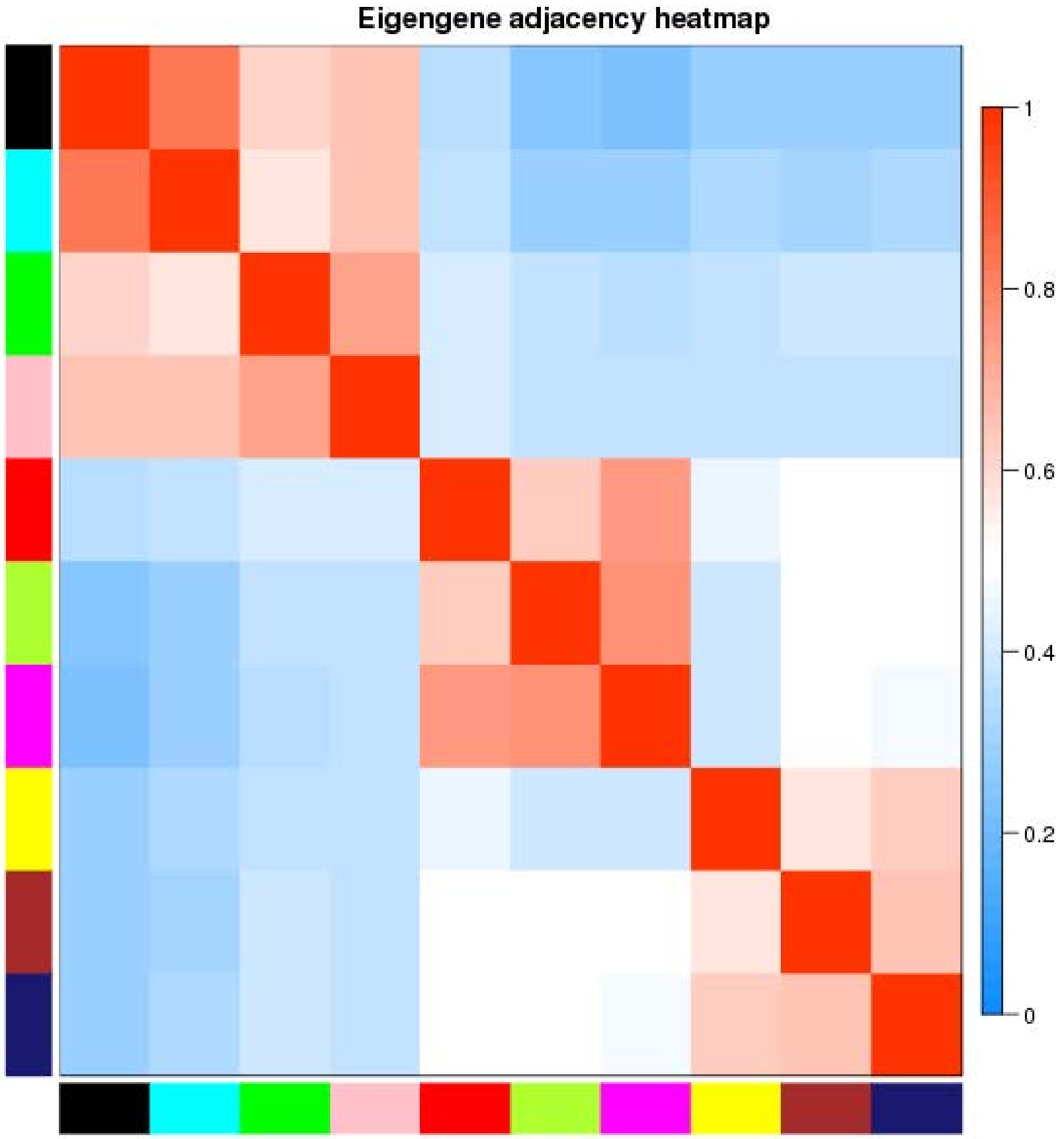

Fig. S14 The percentage of miRNA target isoforms in all isoforms. a) the percentage of miRNA targeted isoforms in each module isoforms. b) the percentage of miRNA targeted isoforms in hub isoforms of each module. c) the χ2 test analysis between the the proportion of isoforms in all modules being targeted by miRNAs and the corresponding proportion of isoforms in hubs

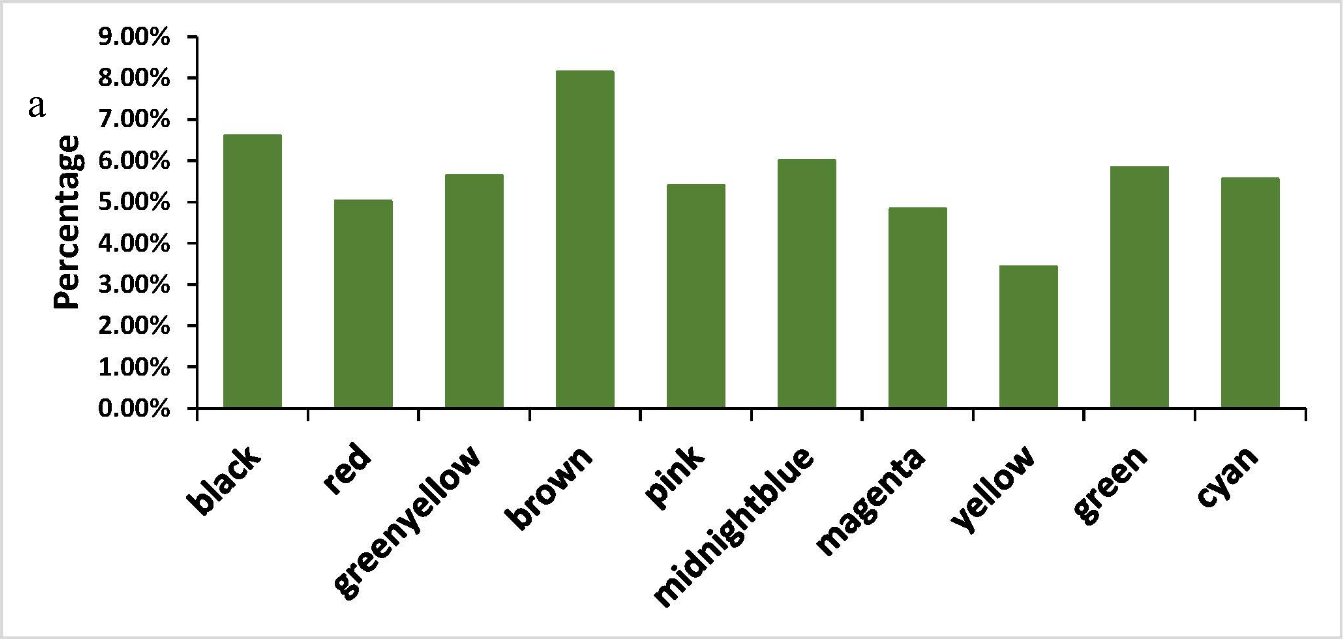

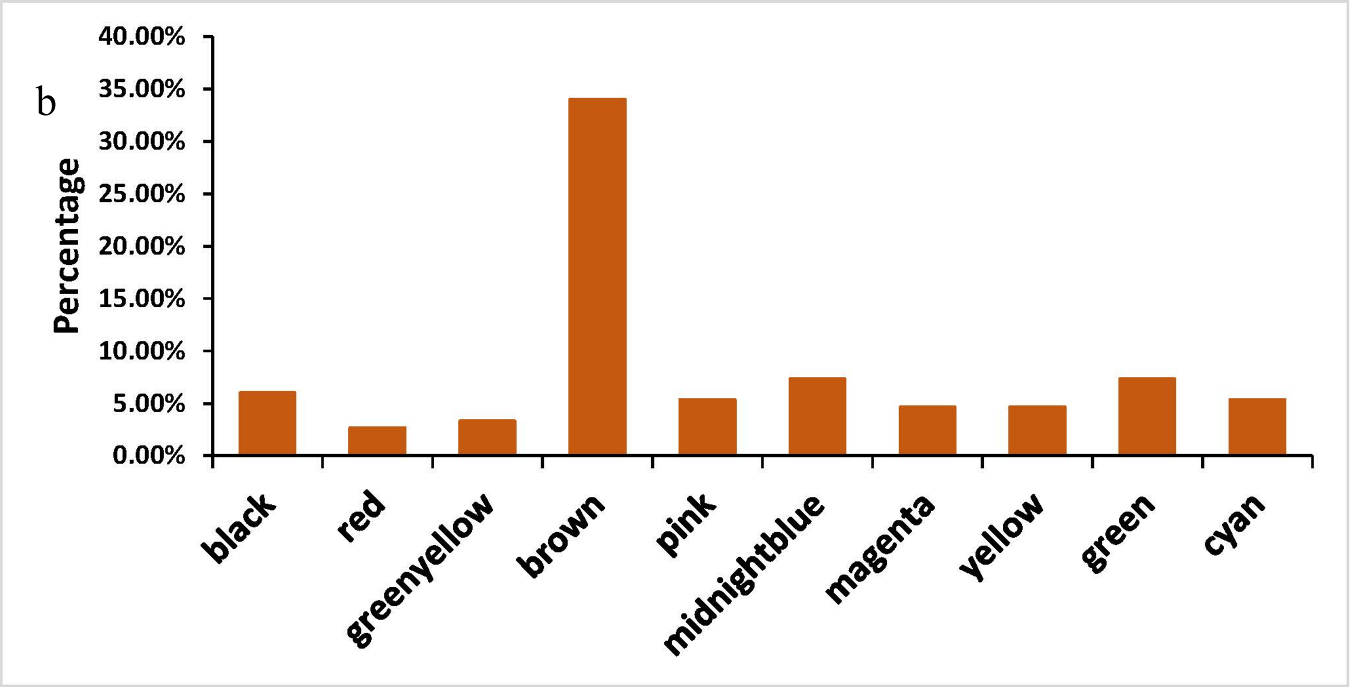

c

module	black	red	greenyellow	brown	pink	midnightblue	magenta	yellow	green	cyan
p-value	0.781	0.213	0.259	1.26E-17	0.975	0.565	0.932	0.450	0.481	0.912

Fig. S15 The isoform module distribution of a IDDM gene “Nn5g29774. The isoforms which are filtered out are transparent linked by imaginary line. The module isoforms are colored in the color of corresponding module. The isoforms targeted by miRNAs are linked by blue line.

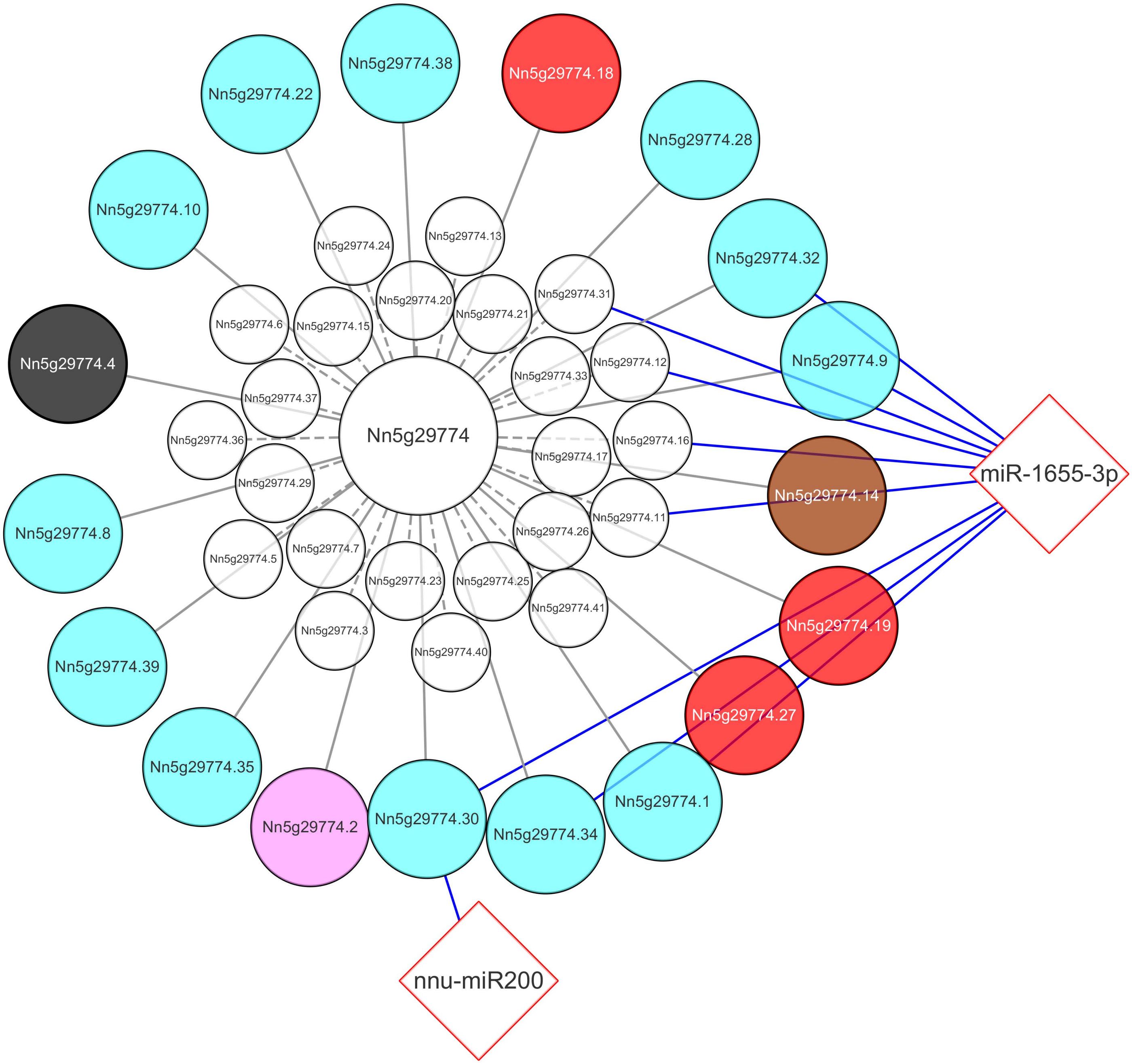

Fig. S16 The homology analyses of filtered isoforms between lotus and rice/Arabidopsis. The inside track is the result of genes which had at least two isoforms mapped to the rice/Arabidopsis. The out track is the result of genes whose isoforms divided into different modules. Gene can be divided into three categories:different isoforms from the same lotus gene with their closest homologs being different genes in rice or Arabidopsis (I); different isoforms from the same lotus gene with their closest homologs being the same one isoform from one gene in rice or Arabidopsis (II); different isoforms from the same lotus gene with their closest homologs being different isoforms from one gene in rice or Arabidopsis (III)

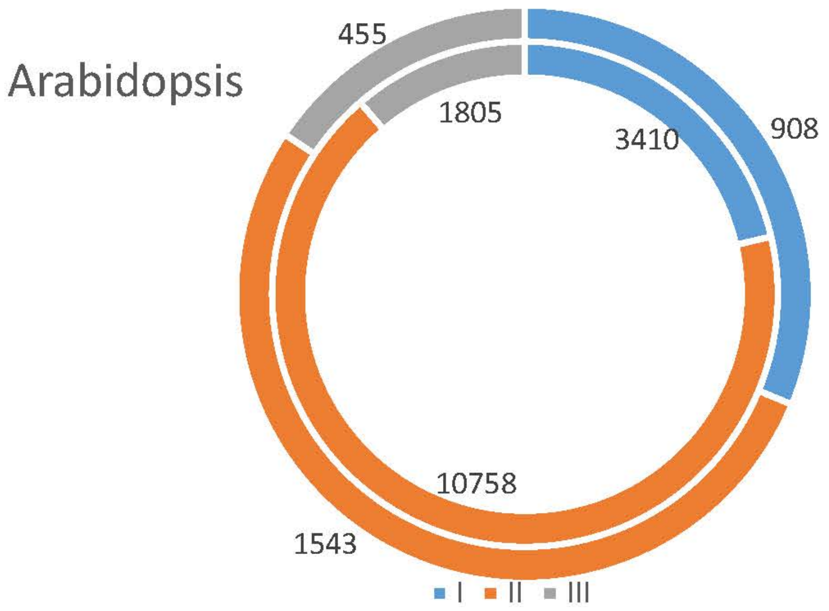

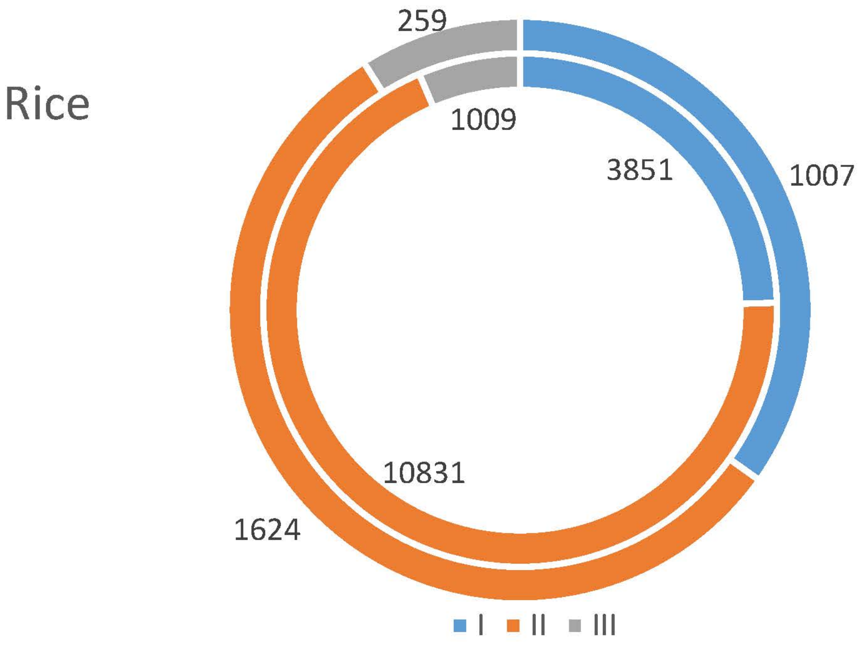

Fig. S17 Plant hormone signal transduction pathway and related miRNAs

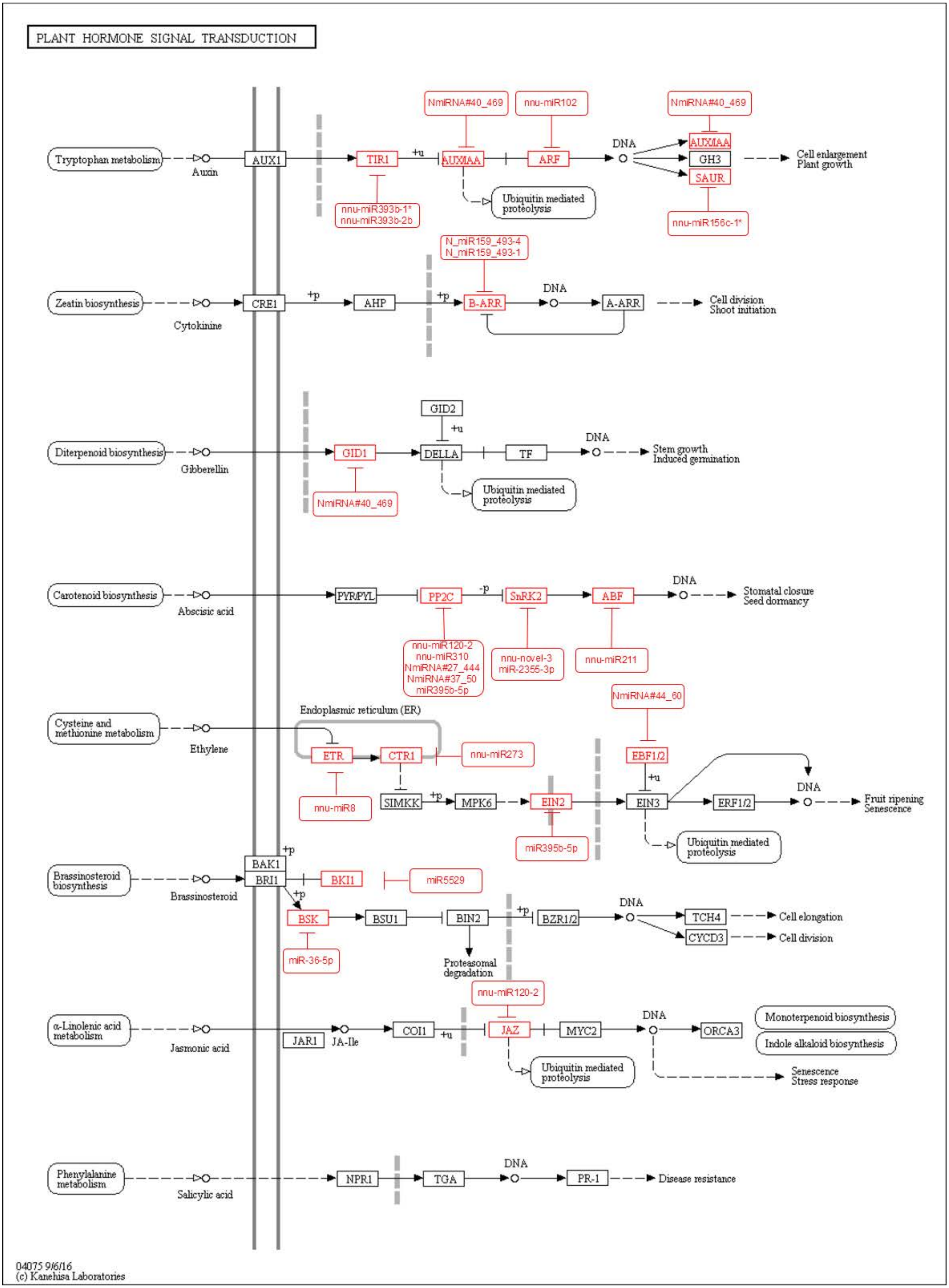

Fig. S18 Plant hormone signal transduction pathway gene and its ortholog genes' isoforms

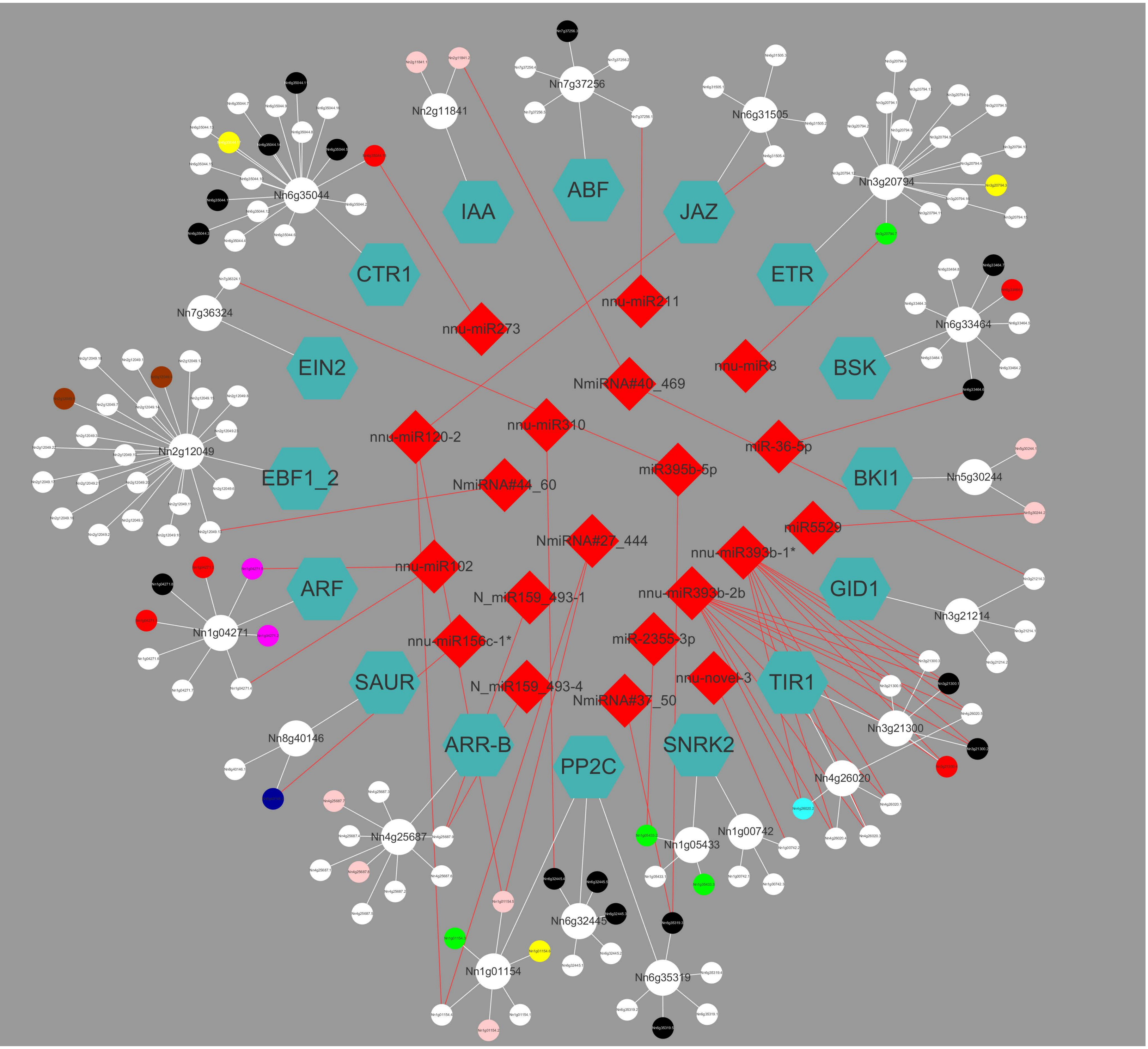

Fig. S19 The expression analysis of abscisic acid-associated genes and related miRNAs expression.

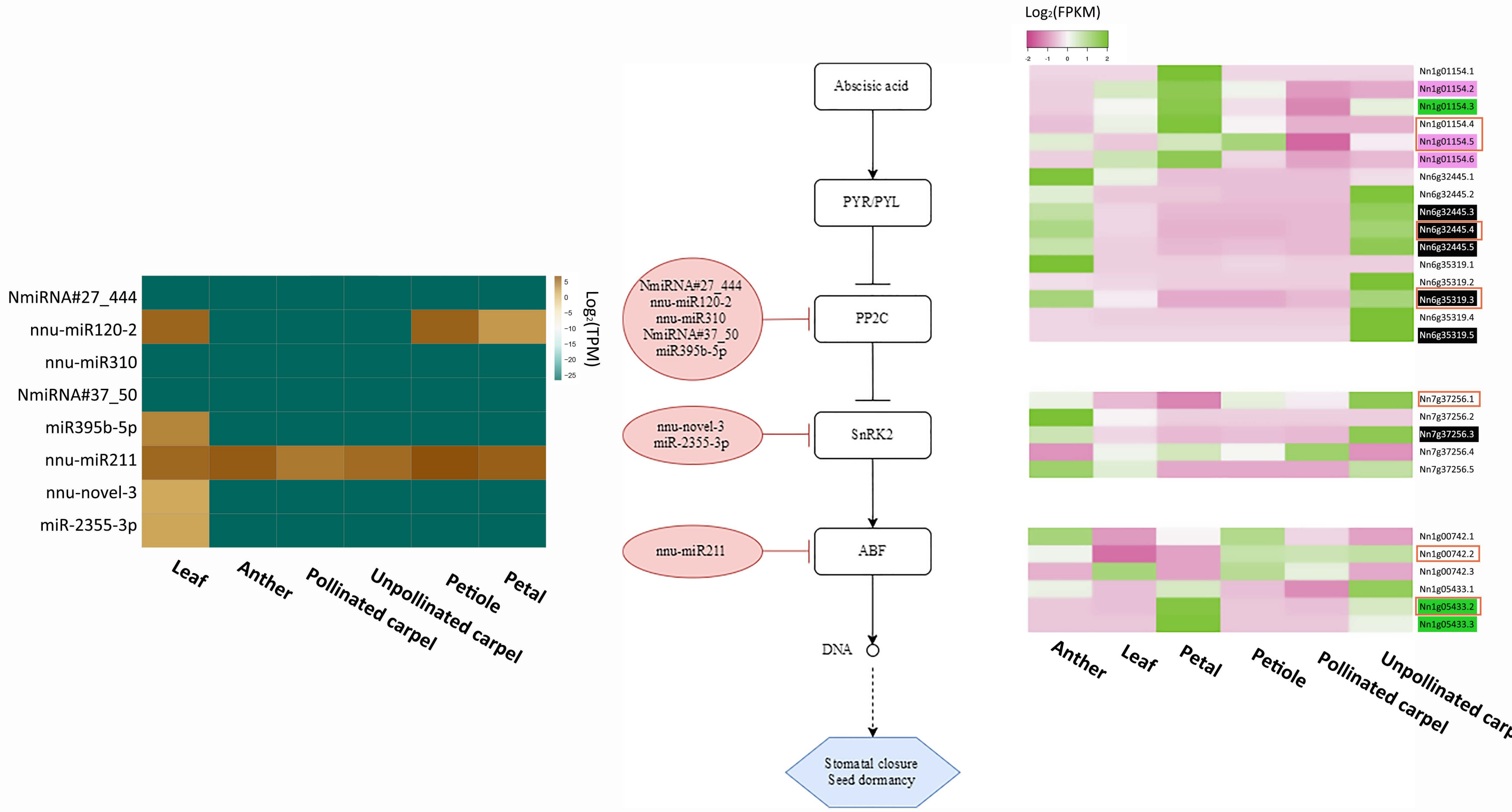

Supplement: Supplementary file 1 — Additional file 1:. Figures S1-S19. [file 12864_2020_6853_MOESM1_ESM.pdf]
